# Amelioration of Post‐Stroke Edema and Microcirculatory Dysfunction via Targeted AQP4 Inhibition While Preserving the Glymphatic System

**DOI:** 10.1002/advs.202520118

**Published:** 2025-12-12

**Authors:** Lei Jin, Zeyu Yang, Boyang Wei, Yu Wu, Longxiang Li, Jiaming Zhou, Xin Zhang, Fa Jin, Shixing Su, Yanchao Liu, Ran Li, Shenquan Guo, Xingwu Liu, Yu Cai, Hong Liu, Min Chen, Wenchao Liu, Chuanzhi Duan, Xifeng Li

**Affiliations:** ^1^ Neurosurgery Center Department of Cerebrovascular Surgery The National Key Clinical Specialty Engineering Research Center of Diagnostic and Therapeutic Technology and Devices for Cerebrovascular Diseases in Ministry of Education Guangdong Provincial Key Laboratory on Brain Function Repair and Regeneration Zhujiang Hospital Institute for Brain Science and Intelligence Zhujiang Hospital, Southern Medical University Guangzhou 510282 China; ^2^ Department of Neurosurgery The Second Affiliated Hospital Jiangxi Medical College Nanchang University Nanchang 330006 China; ^3^ Nanomedicine Innovation Center Erasmus (NICE) Erasmus MC 3015 GD Rotterdam Netherlands; ^4^ State Key Laboratory of Antiviral Drugs Henan University Zhengzhou 450046 China; ^5^ Translational Medical Center of Huaihe Hospital Henan University Kaifeng 475004 China

**Keywords:** glymphatic system, ischemic stroke, microcirculatory dysfunction, nano‐delivery systems, subarachnoid hemorrhage

## Abstract

Cerebral edema and hypoperfusion, hallmark pathologies of both hemorrhagic and ischemic stroke, critically compromise clinical outcomes. Astrocytic aquaporin‐4 (AQP4) not only drives post‐stroke brain edema progression but also maintains the protective clearance function of the glymphatic system. Herein, systemic AQP4 inhibition using TGN‐020 (TGN) paradoxically exacerbates global glymphatic dysfunction despite alleviating cerebral edema and microcirculatory dysfunction following subarachnoid hemorrhage (SAH). To overcome this therapeutic dilemma, an angiopep‐2‐functionalized lipid nanoparticle (A‐LNP) platform enabling lesion‐targeted TGN delivery is engineered. This system reverses the detrimental effects of TGN on the post‐SAH glymphatic system while enhancing the therapeutic benefits of TGN in mitigating cerebral edema and microcirculatory dysfunction. Remarkably, TGN demonstrates multimodal efficacy in ischemic stroke by mitigating the no‐reflow phenomenon, alleviating blood‐brain barrier disruption, and suppressing neuroinflammation. The A‐LNP system retains the protective effects of TGN without compromising global glymphatic function, leading to enhanced therapeutic efficacy. These findings confirm the feasibility of using functional nanoparticles to enhance the protective effects of AQP4 inhibition while minimizing adverse effects on the glymphatic system, offering a promising therapeutic strategy for both stroke subtypes.

## Graphical abstract

Compared to untargeted therapy, the targeted nanocarrier, Angiopep‐2‐conjugated Lipid Nanoparticle (A‐LNP) loaded with TGN‐020 (TGN), selectively accumulated in stroke‐injured regions. It suppressed local aquaporin‐4 (AQP4) overexpression, thereby alleviating cerebral edema and hypoperfusion while preserving global glymphatic clearance.

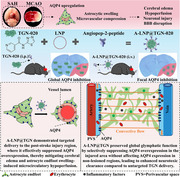

## 1 Introduction

Stroke ranks as the second most common cause of death globally, surpassed only by ischemic heart disease, and is the third leading cause of disability‐adjusted life‐years worldwide.^[^ ^1^ ^]^ This neurological emergency is primarily classified into hemorrhagic stroke, such as subarachnoid hemorrhage (SAH) due to vascular rupture, and ischemic stroke caused by vascular occlusion.^[^ ^2^ ^]^ Despite endovascular therapy, intravenous thrombolysis, and surgical intervention being guideline‐recommended first‐line treatments, their efficacy is constrained by narrow therapeutic time windows, selective patient applicability, and inequitable healthcare access across regions.^[^ ^3^, ^4^ ^]^ Consequently, stroke patients still face high mortality and disability rates.^[^ ^5^ ^]^ Accumulating evidence suggests that post‐stroke cerebral edema and hypoperfusion are key contributors to poor clinical outcomes.^[^ ^6^, ^7^, ^8^, ^9^ ^]^ However, targeted and efficacious therapeutic strategies are still lacking.

Aquaporin‐4 (AQP4) localized at astrocytic endfeet is pivotal in maintaining water homeostasis within the central nervous system.^[^ ^10^ ^]^ Under pathological conditions, AQP4 mediates brain edema progression,^[^ ^11^, ^12^ ^]^ while its genetic knockdown or pharmacological suppression demonstrates significant efficacy in mitigating post‐stroke cerebral edema.^[^ ^13^, ^14^ ^]^ AQP4‐mediated astrocytic endfeet swelling has been reported to impair microvascular perfusion in a mild brain edema model.^[^ ^15^ ^]^ This phenomenon is further amplified in stroke, where endfeet swelling mechanically compresses the microvasculature, resulting in profound microcirculatory hypoperfusion.^[^ ^16^, ^17^ ^]^ A clinical study demonstrates that persistent post‐reperfusion hypoperfusion in infarcted regions correlates strongly with space‐occupying cerebral edema.^[^ ^18^ ^]^ Collectively, these data indicate a pathological coupling between cerebral edema and microcirculatory impairment, wherein both processes mutually reinforce each other, establishing a vicious cycle that potentiates secondary brain injury. These findings suggest that AQP4 inhibition may offer a dual therapeutic advantage by simultaneously addressing both edema formation and hypoperfusion following stroke. Nevertheless, although the neuroprotective effects of AQP4 suppression in reducing stroke‐induced brain edema are well‐documented, its critical involvement in glymphatic waste clearance creates a fundamental therapeutic dilemma. This functional dichotomy urgently demands innovative approaches to reconcile targeted intervention with systemic homeostasis.

The glymphatic system (GS) is a fluid transport network of cerebrospinal fluid (CSF) entering the brain along arterial perivascular spaces, exchanging with interstitial fluid (ISF), ultimately establishing directional clearance of interstitial solutes.^[^ ^19^ ^]^ This CSF‐ISF exchange is critically dependent on the polarized expression of AQP4 at astrocytic endfeet ensheathing cerebral vasculature.^[^ ^20^ ^]^ Compelling evidence from Alzheimer's disease models demonstrates that AQP4 inhibition disrupts glymphatic function and compromises the clearance of pathogenic factors such as tau proteins.^[^ ^21^, ^22^ ^]^ Notably, glymphatic dysfunction has been consistently observed across both hemorrhagic and ischemic stroke subtypes. Restoration of the GS function through reversing AQP4 depolarization has been shown to ameliorate post‐stroke neurological deficits.^[^ ^23^, ^24^ ^]^ These findings reveal a complexity in AQP4 function: while its suppression may attenuate cerebral edema, such intervention concurrently impairs the glymphatic system's clearance capacity. These roles underscore the limitations of untargeted AQP4 modulation as a therapeutic strategy. Consequently, developing spatially targeted delivery systems that selectively enhance drug accumulation in injured brain regions while preserving whole‐brain glymphatic function emerges as an urgent unmet need in stroke therapeutics.

TGN‐020 (TGN), a selective AQP4 inhibitor, significantly reduces brain edema and neurological deficits after ischemic stroke.^[^ ^25^, ^26^ ^]^ However, two critical questions remain unresolved: whether TGN's protective effects could be extended to SAH, and whether it can ameliorate stroke‐mediated microcirculatory hypoperfusion. Notably, existing studies demonstrate that TGN administration impairs GS function in models of traumatic brain injury and intracerebral hemorrhage,^[^ ^27^, ^28^ ^]^ revealing a therapeutic limitation. To address these challenges, we developed an engineered lipid nanoparticle (LNP) platform based on its high target specificity, modifiability, and biocompatibility.^[^ ^29^ ^]^ Low‐density lipoprotein receptor‐related protein 1 (LRP1) is highly expressed in both endothelial cells and astrocytes that constitute the blood‐brain barrier (BBB). The angiopep‐2 peptide, a ligand of LRP1, efficiently facilitates the transport of biomacromolecules across the BBB.^[^ ^30^, ^31^ ^]^ Our LNP system incorporated angiopep‐2 to enhance BBB penetration and improve astrocyte‐targeting specificity.

**Scheme 1:**
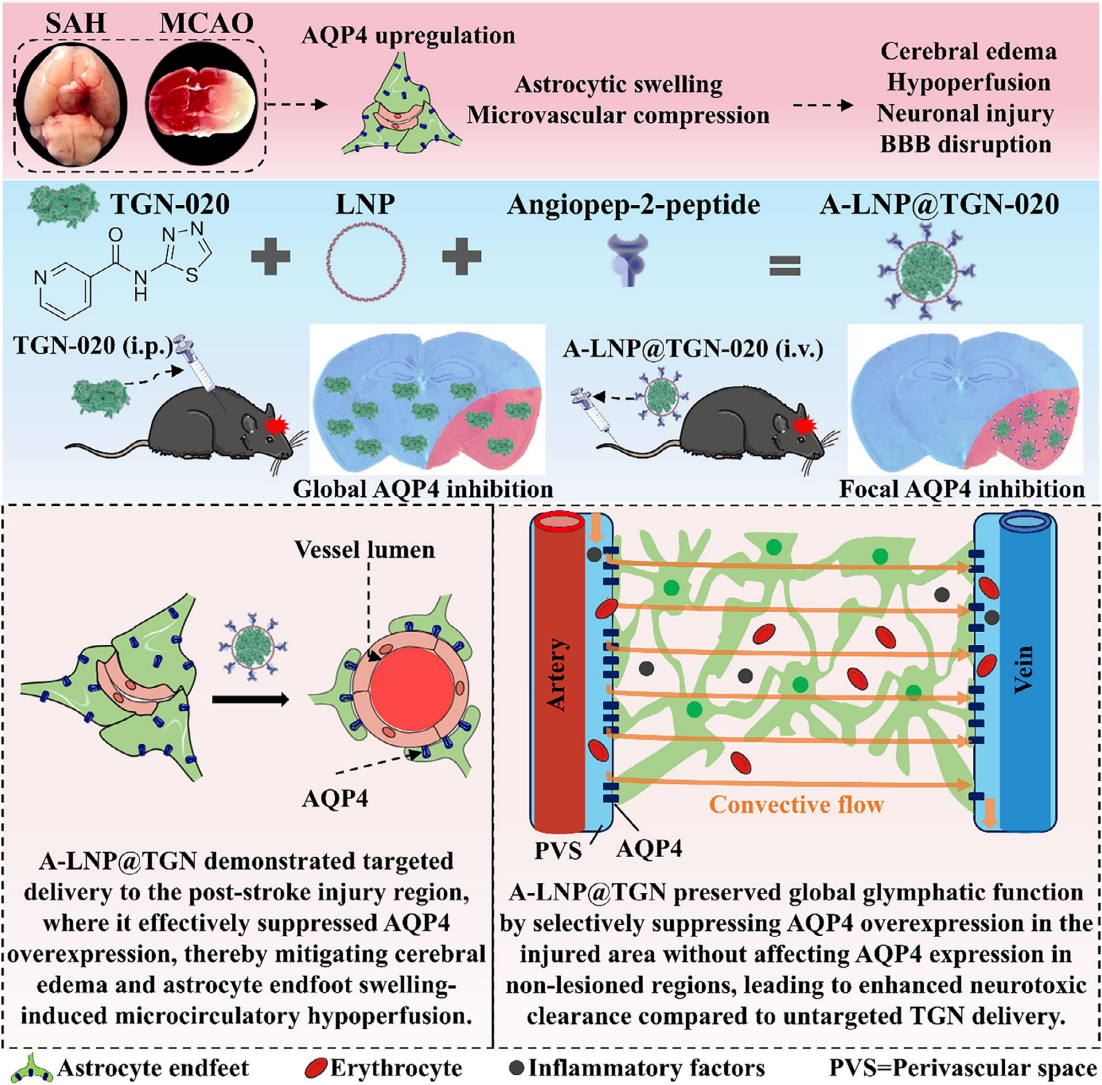
Schematic illustration of A‐LNP@TGN and its therapeutic mechanism in mitigating stroke‐induced brain injury.

In this study, we first elucidated TGN's dual effects on SAH‐induced brain injury and GS dysfunction, establishing the rationale for targeted delivery. Building on these findings, we engineered multifunctional nanoparticles (A‐LNP@TGN) by integrating angiopep‐2 peptide and LNP to achieve precise delivery of TGN to stroke‐affected brain regions (**Scheme** **1**). Through comprehensive in vivo evaluations across both hemorrhagic and ischemic stroke models, we systematically assessed the therapeutic impact of A‐LNP@TGN on key pathological outcomes: cerebral edema formation, microcirculation disturbance, and neurological recovery. Notably, A‐LNP@TGN selectively accumulated in injured areas, ameliorated cerebral edema and microcirculatory hypoperfusion, and preserved whole‐brain glymphatic clearance capacity.

## 2 Results

### 2.1 TGN Ameliorates SAH‐Induced Cerebral Edema and Microcirculatory Impairment

**Figure 1:**
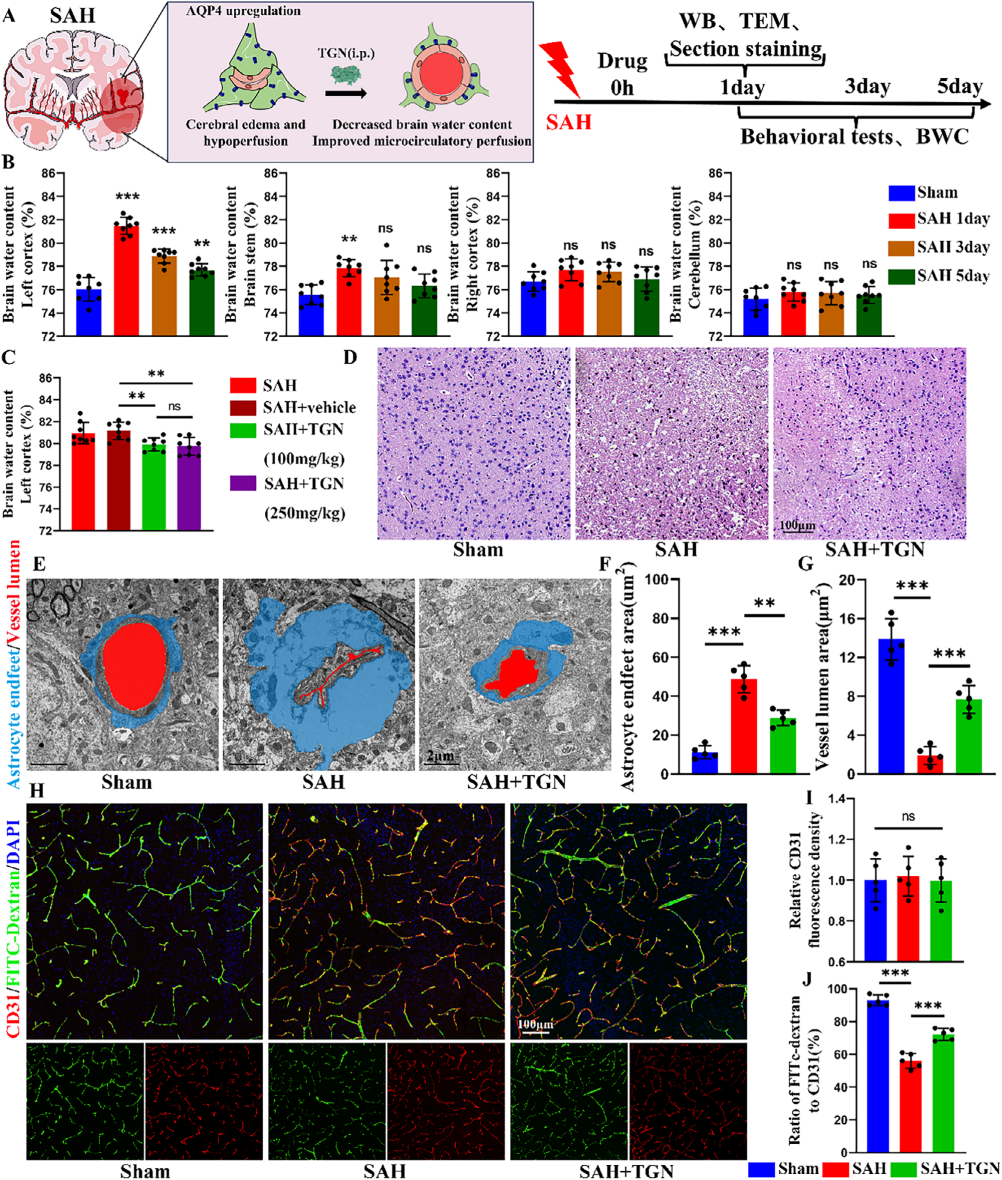
TGN ameliorates SAH‐induced cerebral edema and microcirculatory impairment. A) Schematic diagram of the mechanism and experimental flow chart. B) Quantification of brain water content of different parts after SAH. C) Quantification of brain water content in the left cortex between groups 24 h after SAH (*n* = 8). D) Representative H&E staining images of brain edema for each group (*n* = 5). E–G) Representative TEM images of the ultrastructure of microvessels for each group and corresponding statistical results (*n* = 5). H–J) Representative immunofluorescence images of microcirculation in different groups and corresponding statistical results (*n* = 5). Data represent the mean ± SD, ns = no significant, ^**^ *p* < 0.01, ^***^ *p* < 0.001.

We performed a series of experiments to evaluate the therapeutic effects of TGN on SAH‐induced brain injury (**Figure** **1**A). Initial characterization revealed that post‐SAH brain edema exhibited left cortical predominance, peaking at day 1 and gradually subsiding thereafter, though significant swelling persisted through day 5 (Figure 1B). Based on these findings, we focused subsequent analyses on the ipsilateral temporal cortex adjacent to the hemorrhage site. Western blot analysis demonstrated significant upregulation of AQP4 expression following SAH (Figure S1, Supporting Information). Guided by previous studies,^[^ ^21^, ^32^ ^]^ we evaluated two concentrations of intraperitoneally administered TGN. The quantitative assessment demonstrated comparable efficacy between low‐ and high‐dose TGN in attenuating SAH‐induced cerebral edema (Figure 1C), prompting the selection of the lower dose for subsequent experiments to minimize potential off‐target effects. Western blot showed that TGN significantly suppressed SAH‐induced AQP4 upregulation (Figure S2, Supporting Information). Hematoxylin and eosin (H&E) staining exhibited reduced edema‐associated vacuolation in the injured area following TGN treatment (Figure 1D). Transmission electron microscopy (TEM) demonstrated that TGN intervention markedly ameliorated SAH‐induced pathology, including astrocytic endfeet swelling and vascular lumen constriction (Figure 1E–G). Notably, while immunofluorescence staining showed no significant change in vascular density, TGN administration markedly attenuated SAH‐induced microcirculatory hypoperfusion (Figure 1H–J).

### 2.2 TGN Aggravates Post‐SAH Glymphatic Clearance Dysfunction

**Figure 2:**
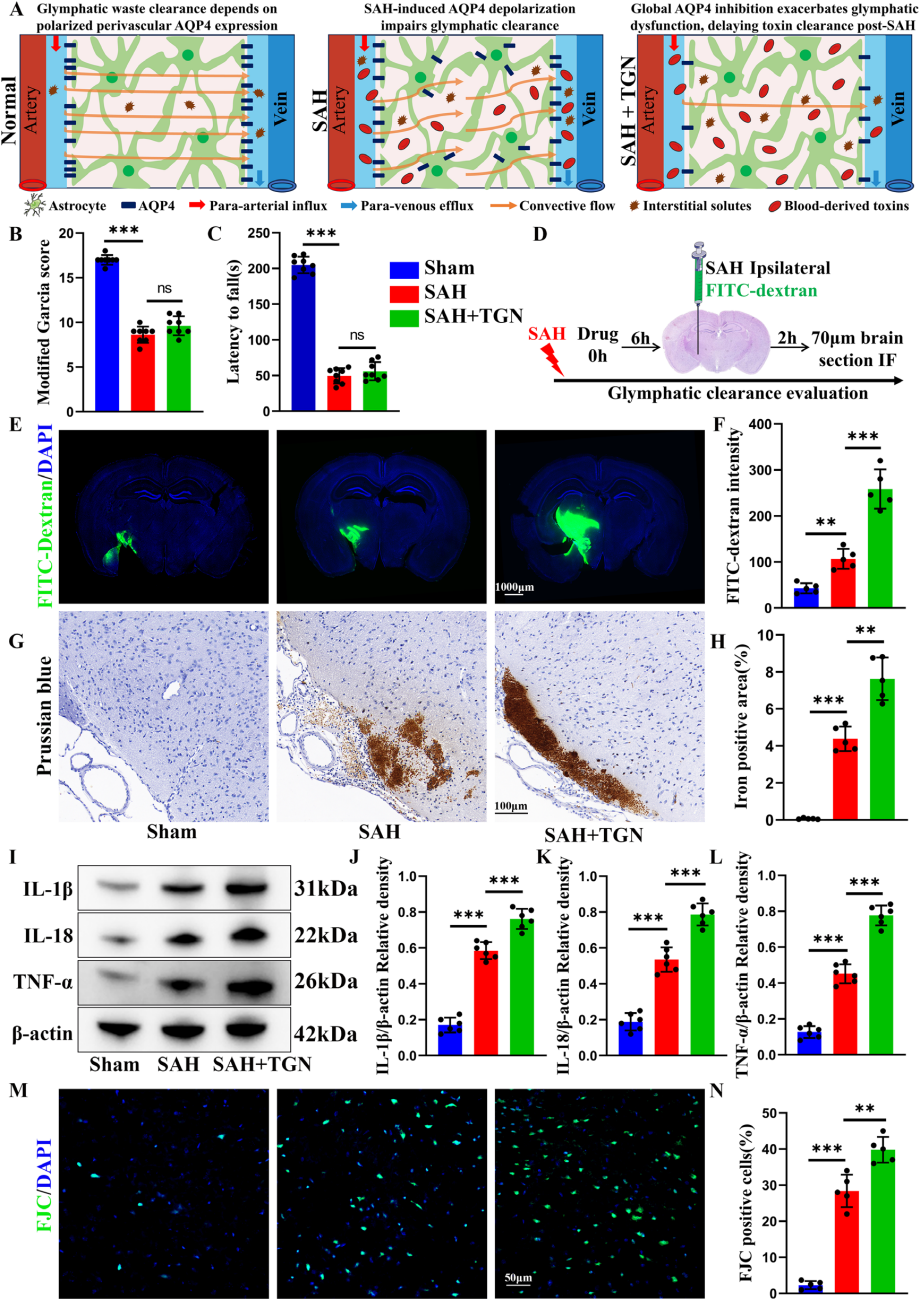
TGN aggravates post‐SAH glymphatic clearance dysfunction. A) Schematic diagram illustrating the glymphatic system under different conditions. B) Modified Garcia scores for each group (*n* = 8). C) Rotarod test for each group (*n* = 8). D) Flow chart of stereotaxic injection. E,F) Representative immunofluorescence images of glymphatic clearance and statistical result (*n* = 5). G,H) Representative Prussian blue staining images and statistical results (*n* = 5). I–L) Representative western blot images of pro‐inflammatory cytokines and statistical results (*n* = 6). M,N) Representative FJC staining images for each group and statistical result (*n* = 5). Data represent the mean ± SD, ns = no significant, ^**^ *p* < 0.01, ^***^ *p* < 0.001.

The schematic illustrates that SAH impairs glymphatic system function, and this compromised glymphatic function is further exacerbated by non‐targeted TGN. (**Figure** **2**A). Contrary to our expectations, TGN treatment did not exhibit significant alleviation of SAH‐induced neurological deficits (Figure 2B,C). Given that AQP4 plays a critical role in glymphatic system homeostasis, and that glymphatic clearance function has a protective effect against brain injury after SAH, the impact of the AQP4‐selective inhibitor TGN on the glymphatic system following SAH remains unclear. To elucidate these paradoxical findings, we evaluated glymphatic system function through stereotaxic delivery of FITC‐dextran following SAH induction (Figure 2D). Our results revealed significant impairment of FITC‐dextran clearance in SAH animals relative to sham controls, with TGN treatment further aggravating this clearance dysfunction (Figure 2E,F). These findings demonstrated that TGN pronouncedly compromises glymphatic clearance function after SAH, likely resulting in the accumulation of neurotoxic metabolites. Prussian blue staining showed increased cerebral iron deposition in TGN‐treated SAH mice (Figure 2G,H), suggesting compromised clearance of erythrocytes and their hemoglobin degradation products. Western blot analysis further demonstrated that TGN amplified post‐SAH neuroinflammation, as evidenced by elevated levels of pro‐inflammatory cytokines including IL‐1β, IL‐18, and TNF‐α (Figure 2I–L). Consistent with these findings, Fluoro‐Jade C (FJC) staining demonstrated a marked increase in neuronal degeneration with TGN treatment compared to SAH controls (Figure 2M,N). These results collectively provided a mechanistic explanation for the absence of neurological recovery with TGN treatment after SAH.

### 2.3 Preparation and Characterization of A‐LNP@TGN

**Figure 3:**
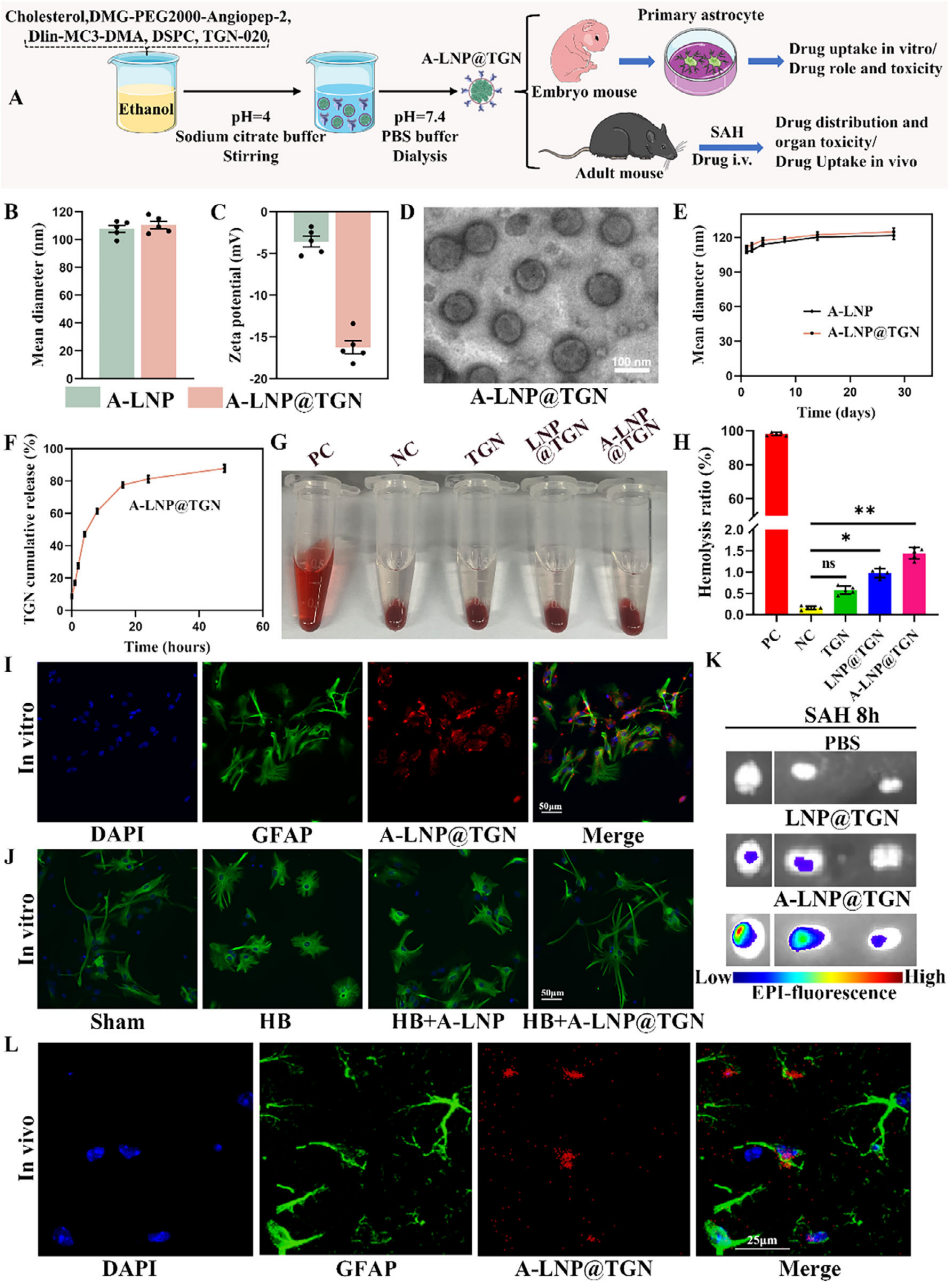
Characterization of A‐LNP@TGN. A) Experimental flow chart for preparation and verification of A‐LNP@TGN. B) Particle size distribution by DLS (*n* = 5). C) Zeta potential measurement (*n* = 5). D) TEM image illustrating the morphology of A‐LNP@TGN. E) Long‐term stability of A‐LNP and A‐LNP@TGN at 4 °C (*n* = 5). F) In vitro release profile of TGN in A‐LNP@TGN (*n* = 3). G,H) Hemolysis test and statistical result (*n* = 5). I) Cellular uptake of A‐LNP@TGN (red) in astrocytes (green). Nuclei are blue (DAPI) (*n* = 5). J) Astrocyte morphology (GFAP, green) across treatment groups (*n* = 5). HB = hemoglobin. K) Representative in vivo imaging of DiR‐labeled nanoparticles in the brain after SAH 8 h for each group (*n* = 5). L) Representative immunofluorescence images of uptake of A‐LNP@TGN by astrocyte in vivo (*n* = 5). Data represent the mean ± SD, ns = no significant, ^*^ *p* < 0.05, ^**^ *p* < 0.01.

In order to overcome this therapeutic dilemma, the LNP‐based delivery TGN system was synthesized. To evaluate the cellular uptake and systemic distribution of our nanoparticle system, primary astrocytes were isolated from neonatal mice within 24 h of birth, and an SAH model was generated in adult male mice for further experiments (**Figure** **3**A). We selected LNPs composed of the ionizable lipid Dlin‐MC3‐DMA to load TGN through hydrophilic‐hydrophobic interactions while incorporating the angiopep‐2 peptide to achieve BBB penetration and astrocyte targeting in the brain lesion site. Dynamic light scattering (DLS) analysis demonstrated that the average particle sizes of A‐LNP and A‐LNP@TGN were 108 ± 6 nm and 110 ± 6 nm, both exhibiting narrow particle size distributions (Figure 3B; Figure S3, Supporting Information). Zeta potentials of A‐LNP and A‐LNP@TGN were ‐3.6 ± 1.5 mV and ‐16.2 ± 1.8 mV (Figure 3C), which are favorable for prolonging the circulation time of the particles in vivo. Additionally, TEM images confirmed that A‐LNP@TGN possessed spherical nanoparticle morphology (Figure 3D). Subsequently, the storage stability of the particles and the in vitro release behavior of TGN were investigated. The results showed that the A‐LNP and A‐LNP@TGN particle sizes did not change significantly during the 30‐day observation period, and the loading of TGN did not affect the stability of the particles (Figure 3E). The in vitro release profile of TGN demonstrated that A‐LNP@TGN exhibited an encapsulation efficiency of 91.4 ± 2.4% for TGN. ≈61.3% of TGN was released from the particles within 8 h, followed by a slow‐release phase, with more than 80% release achieved after 24 h (Figure 3F). Pharmacokinetic analysis from the in vivo study revealed that A‐LNP@TGN exhibited an AUC of 70.28 ± 1.94 µg mL^−1^∙h, an elimination half‐life of 12.27 h, and a clearance of 0.34 Ml h^−1^ (Figure S4 Supporting Information). These findings indicated that A‐LNP@TGN maintained a prolonged circulation time in the bloodstream, thereby enhancing its passive targeting capability for drug delivery. Together, these results indicate that A‐LNP@TGN are spherical nanoparticles with excellent stability and are capable of achieving controlled release of TGN under physiological conditions.

### 2.4 Cellular Uptake and Bio Distribution Profile of A‐LNP@TGN

Cell Counting Kit‐8 (CCK‐8) verified the safety of A‐LNP@TGN in vitro and screened the optimal dose for further experiments (Figure S5, Supporting Information). In addition, the hemolysis test confirmed that nanoparticles are safe for blood cells (Figure 3G,H). Blood routine examination and serum biochemistry analysis revealed no abnormal blood and serum parameters (Figure S6, Supporting Information). H&E staining demonstrated no pathological changes in major organs (Figure S7 Supporting Information). The data indicated good biocompatibility of the material in vivo. Subsequent experiments provided conclusive verification of cellular uptake. The flow cytometry results showed a pronounced increase in fluorescence intensity in astrocyte cells in the A‐LNP@TGN group compared to the LNP@TGN group without modification of the targeting peptide (Figure S8 Supporting Information). Immunofluorescence also confirmed the uptake of A‐LNP@TGN by astrocytes in vitro, and A‐LNP@TGN could reduce hemin‐induced astrocyte swelling (Figure 3I,J). Then, the ability of A‐LNP@TGN to penetrate the BBB was evaluated. In vitro BBB model demonstrated DiR‐labeled‐A‐LNP@TGN exhibiting more pronounced fluorescence intensities in the lower chamber compared to DiR‐labeled‐LNP without angiopep‐2 peptide (Figure S9 Supporting Information). Subsequently, we injected DiR‐labeled A‐LNP@TGN via the tail vein after SAH induction to assess its distribution in vivo. A‐LNP@TGN showed higher accumulation in the ipsilateral cortex than non‐targeted LNP@TGN (Figure 3K). Detectable signals were also found in hepatic and renal tissues (Figure S10 Supporting Information). Furthermore, immunofluorescence analysis demonstrated that A‐LNP@TGN was successfully taken up by astrocytes in vivo, which was consistent with the in vitro observations (Figure 3L).

### 2.5 A‐LNP@TGN Mitigates SAH‐Induced Brain Injury while Preserving Glymphatic System Function

**Figure 4:**
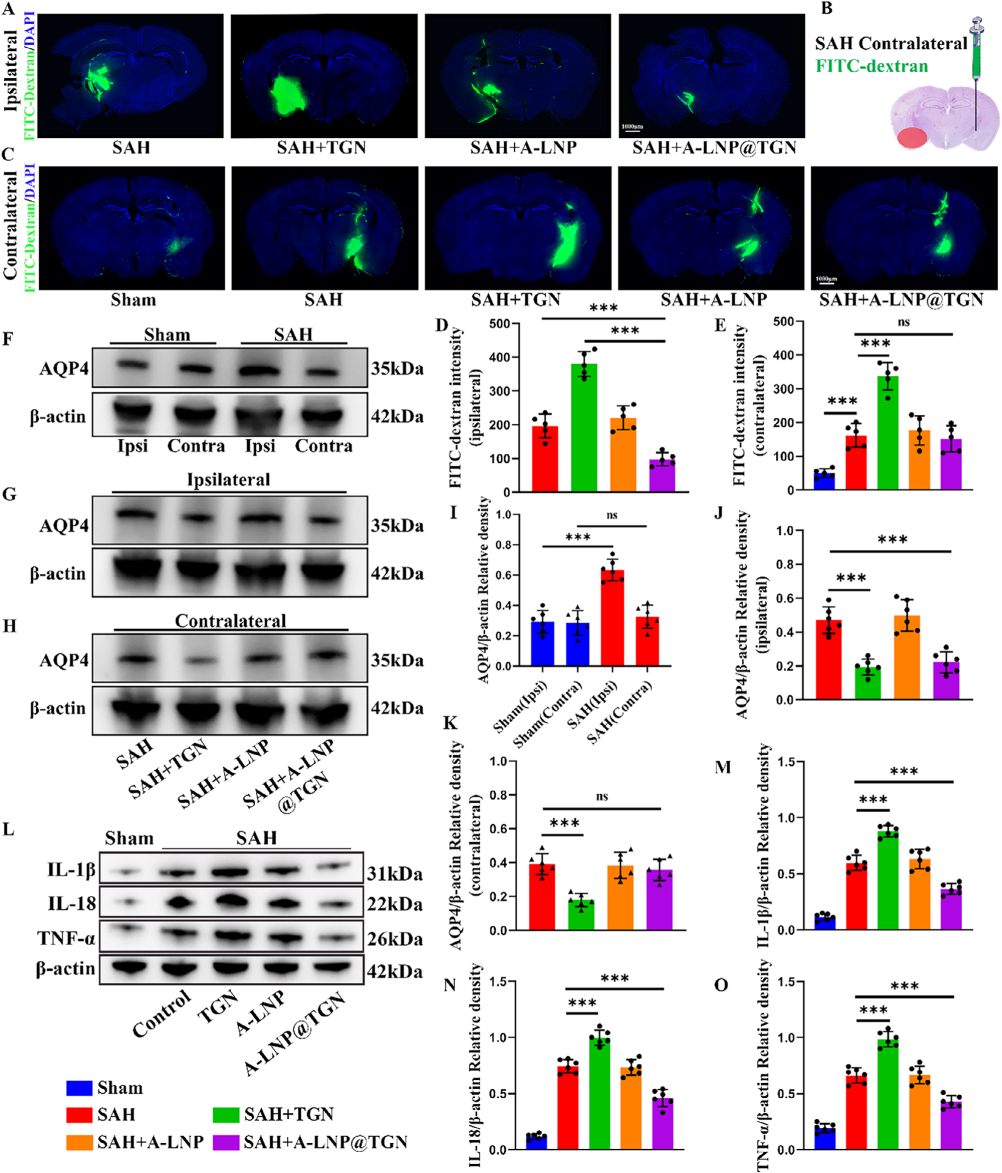
A‐LNP@TGN preserves glymphatic clearance, whereas TGN impairs it. A,C–E) Representative immunofluorescence images of glymphatic clearance and statistical results (*n* = 5). B) Flow chart of stereotaxic injection in the contralateral hemisphere. F–K) Representative western blot images of AQP4 across different groups and statistical results (*n* = 6). L–O) Representative western blot images of inflammatory factors across different groups and statistical results (*n* = 6). And the color scheme in the figure corresponds to different experimental groups. Data represent the mean ± SD, ns = no significant, ^***^ *p* < 0.001.

Our in vivo studies demonstrated that A‐LNP@TGN provided dual therapeutic benefits by simultaneously alleviating SAH‐induced brain injury and preserving glymphatic clearance function. Compared to non‐targeted TGN, A‐LNP@TGN not only reversed the detrimental effects of TGN on glymphatic clearance but also enhanced the removal of FITC‐dextran in the ipsilateral hemisphere after SAH. Furthermore, while SAH resulted in delayed FITC clearance in the contralateral cortex—an effect exacerbated by TGN—A‐LNP@TGN did not further inhibit FITC clearance in this region (**Figure** **4**A–E). Western blot analysis revealed a significant upregulation of AQP4 expression in the ipsilateral hemisphere following SAH, whereas levels remained largely unaltered in the contralateral side. TGN administration suppressed AQP4 expression in both hemispheres after SAH. Most importantly, A‐LNP@TGN treatment effectively normalized the elevated AQP4 expression in the injured hemisphere without disrupting its physiological levels in the unaffected region (Figure 4F–K). Moreover, in contrast to TGN, A‐LNP@TGN significantly attenuated SAH‐induced neuroinflammation, as indicated by a pronounced reduction in pro‐inflammatory cytokine expression (Figure 4L–O). Collectively, in contrast to TGN, which worsened glymphatic dysfunction and amplified neuroinflammation after SAH, A‐LNP@TGN selectively suppressed AQP4 overexpression in the injured hemisphere without altering its physiological levels in healthy tissue. This preservation of glymphatic function facilitated the clearance of neurotoxic substances, leading to a reduction in neuroinflammation.

**Figure 5:**
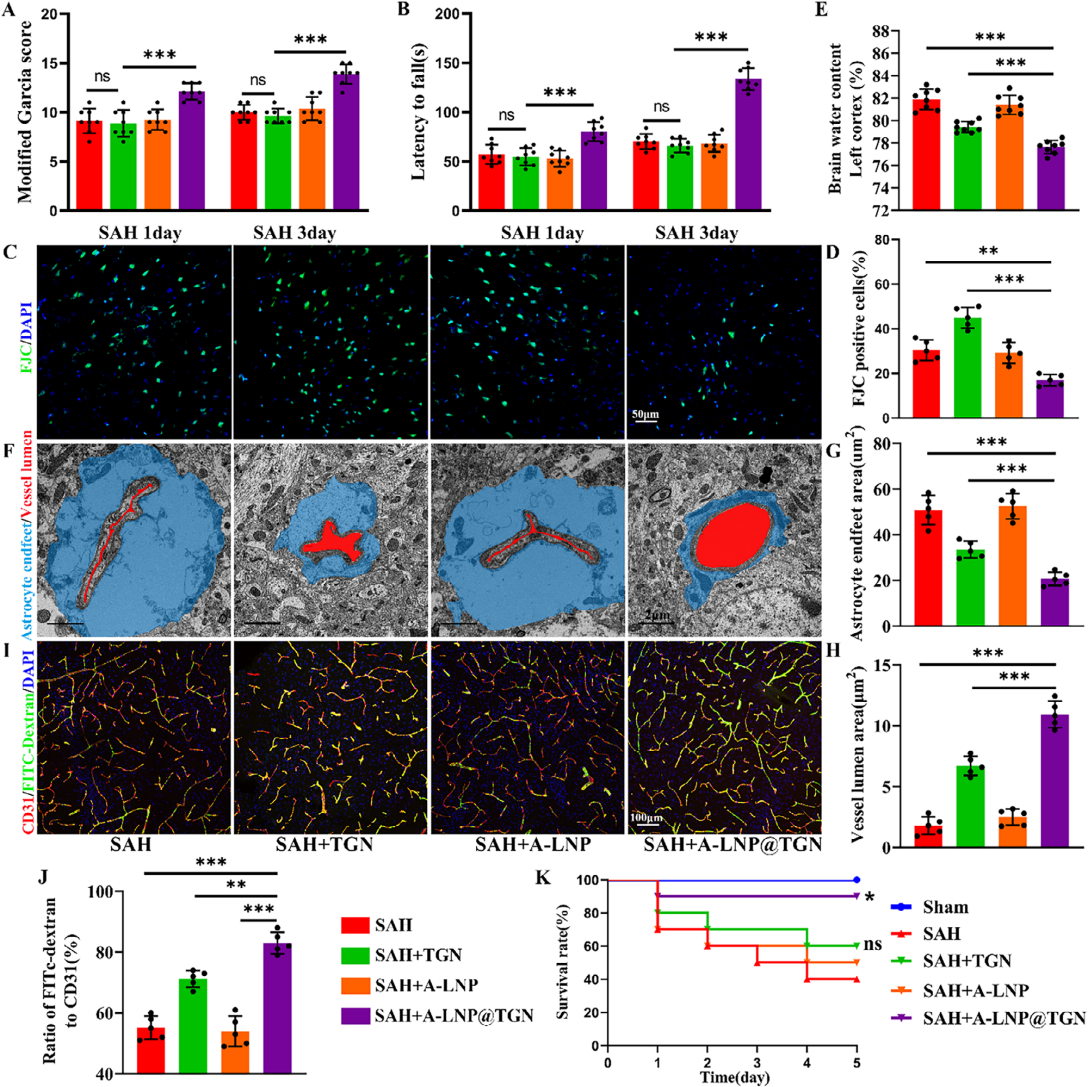
A‐LNP@TGN provides superior therapeutic efficacy against SAH‐induced brain injury compared to TGN. A) Modified Garcia scores for each group (*n* = 8). B) Rotarod test for each group (*n* = 8). C,D) FJC staining images for each group and statistical result (*n* = 5). E) Quantification of brain water content in the left cortex between groups 24 h after SAH (*n* = 8). F–H) TEM images of the ultrastructure of microvessels for each group and statistical results (*n* = 5). I,J) Immunofluorescence images of microcirculation in different groups and statistical results (*n* = 5). K) Survival rate of each group (*n* = 10; vs SAH). Data represent the mean ± SD, ns = no significant, ^*^ *p* < 0.05, ^**^ *p* < 0.01, ^***^ *p* < 0.001.

Neurological evaluation confirmed that A‐LNP@TGN treatment ameliorated SAH‐induced neurological deficits (**Figure** **5**A,B). This protective effect was further supported by FJC staining, which revealed a marked reduction in neuronal degeneration in the SAH + A‐LNP@TGN group compared to the SAH group (Figure 5C,D). The LNP‐based delivery system also enhanced the therapeutic efficacy of TGN in mitigating cerebral edema and microcirculatory impairment. Specifically, A‐LNP@TGN more effectively reduced cerebral edema than TGN alone (Figure 5E) and demonstrated superior alleviation of post‐SAH astrocytic endfeet swelling and microvascular lumen compression (Figure 5F–H). Immunofluorescence analysis confirmed no significant intergroup differences in vascular density (Figure S11, Supporting Information), yet A‐LNP@TNG treatment resulted in better recovery of microcirculatory perfusion compared to TGN alone (Figure 5I,J). Both TGN and A‐LNP@TGN attenuated SAH‐associated weight loss, though the LNP‐based formulation promoted more substantial weight recovery (Figure S12, Supporting Information). Importantly, A‐LNP@TGN improved survival rates after SAH, an effect not observed with TGN monotherapy (Figure 5K). In summary, A‐LNP@TGN attenuated SAH‐induced brain injury by reducing cerebral edema, improving microcirculatory function, and maintaining glymphatic clearance of neurotoxic metabolites.

### 2.6 A‐LNP@TGN Enhances TGN's Neuroprotective Effects Against Ischemic Brain Damage

**Figure 6:**
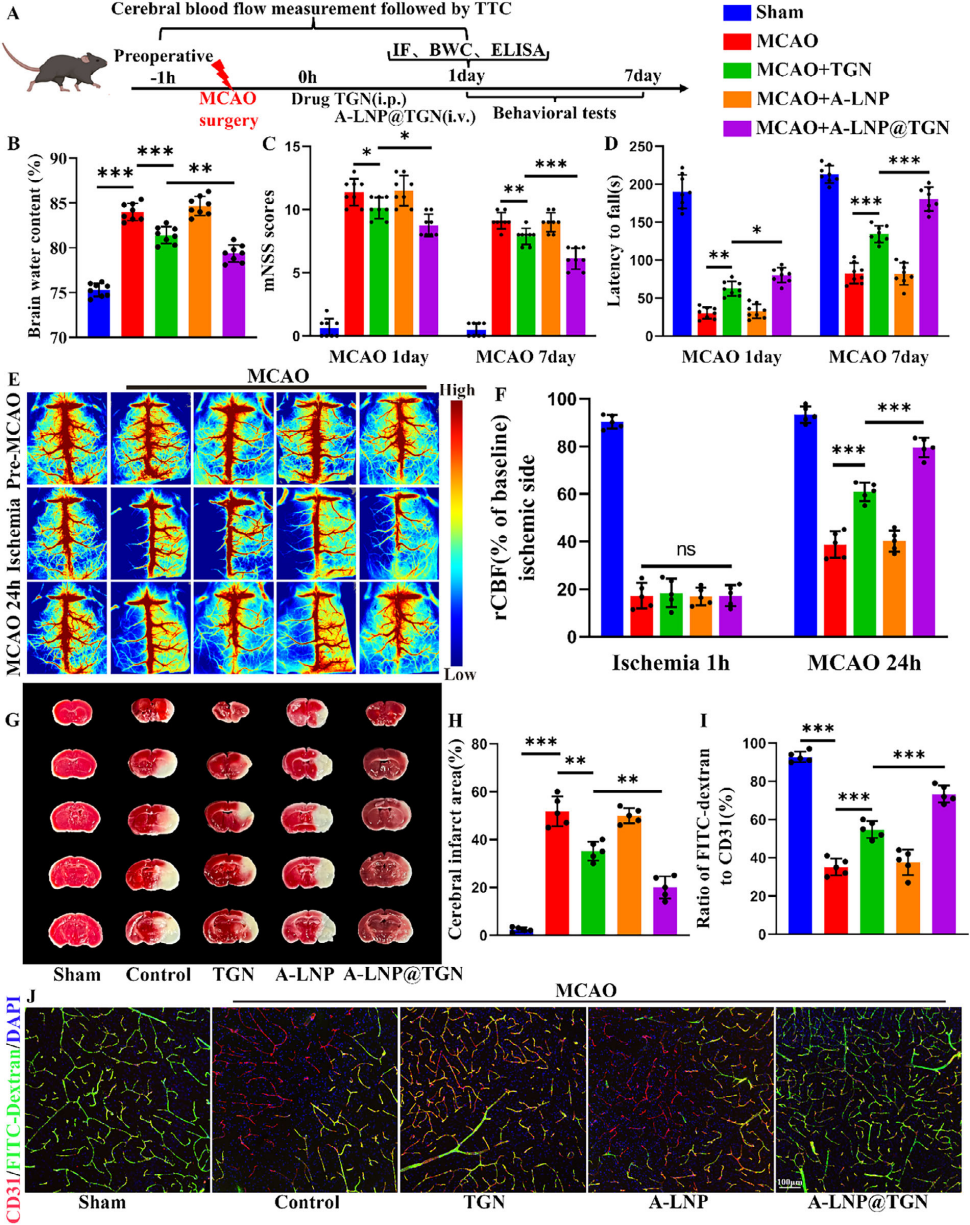
A‐LNP@TGN enhances TGN's neuroprotective effects against ischemic hypoperfusion. A) The experimental flow chart and the corresponding figure note the color of different groups. B) Quantification of brain water content in the ipsilateral hemisphere between groups 24 h after MCAO (*n* = 8). C) Modified Neurological Severity Score (mNSS) test for each group (*n* = 8). D) Rotarod test for each group (*n* = 8). E,F) Laser speckle images of different groups between three time points, including pre‐MCAO, ischemia 1 h, and MCAO 24 h (*n* = 5). G,H) TTC results for each group and statistical result (*n* = 5). I,J) Immunofluorescence images of microcirculation in different groups and statistical result (*n* = 5). Data represent the mean ± SD, ns = no significant, ^*^ *p* < 0.05, ^**^ *p* < 0.01, ^***^ *p* < 0.001.

Building upon established evidence of neuroprotective effects of TGN administration through intraperitoneal injection in ischemic stroke models using transient middle cerebral artery occlusion (MCAO), we investigated whether angiopep‐2‐modified LNP encapsulation could enhance its therapeutic efficacy via intravenous delivery (**Figure** **6**A). Our results demonstrated that A‐LNP@TGN provided significantly greater protection against stroke‐induced brain injury compared to non‐targeted TGN, as evidenced by markedly attenuated cerebral edema and improved neurological recovery (Figure 6B–D). Expanding on our previous findings of A‐LNP@TGN's benefits in subarachnoid hemorrhage, we evaluated its impact on post‐ischemic hemodynamics. While laser speckle imaging revealed comparable acute hypoperfusion at ischemia 1 h across different MCAO groups, both TGN and A‐LNP@TGN groups showed substantial cerebral blood flow restoration compared to the MCAO group at 24 h post‐ischemia, with A‐LNP@TGN exhibiting the best restoration of blood perfusion (Figure 6E,F). 2,3,5‐triphenyltetrazolium chloride (TTC) staining corroborated these findings, with the LNP‐based TGN treatment showing superior efficacy in reducing infarct volume compared to other groups (Figure 6G,H). Immunofluorescence staining revealed no significant change in vascular density among groups (Figure S13, Supporting Information). Further analysis confirmed that TGN alleviated the no‐reflow phenomenon in the ischemic region, with A‐LNP@TGN providing more pronounced microcirculatory perfusion (Figure 6I,J).

**Figure 7:**
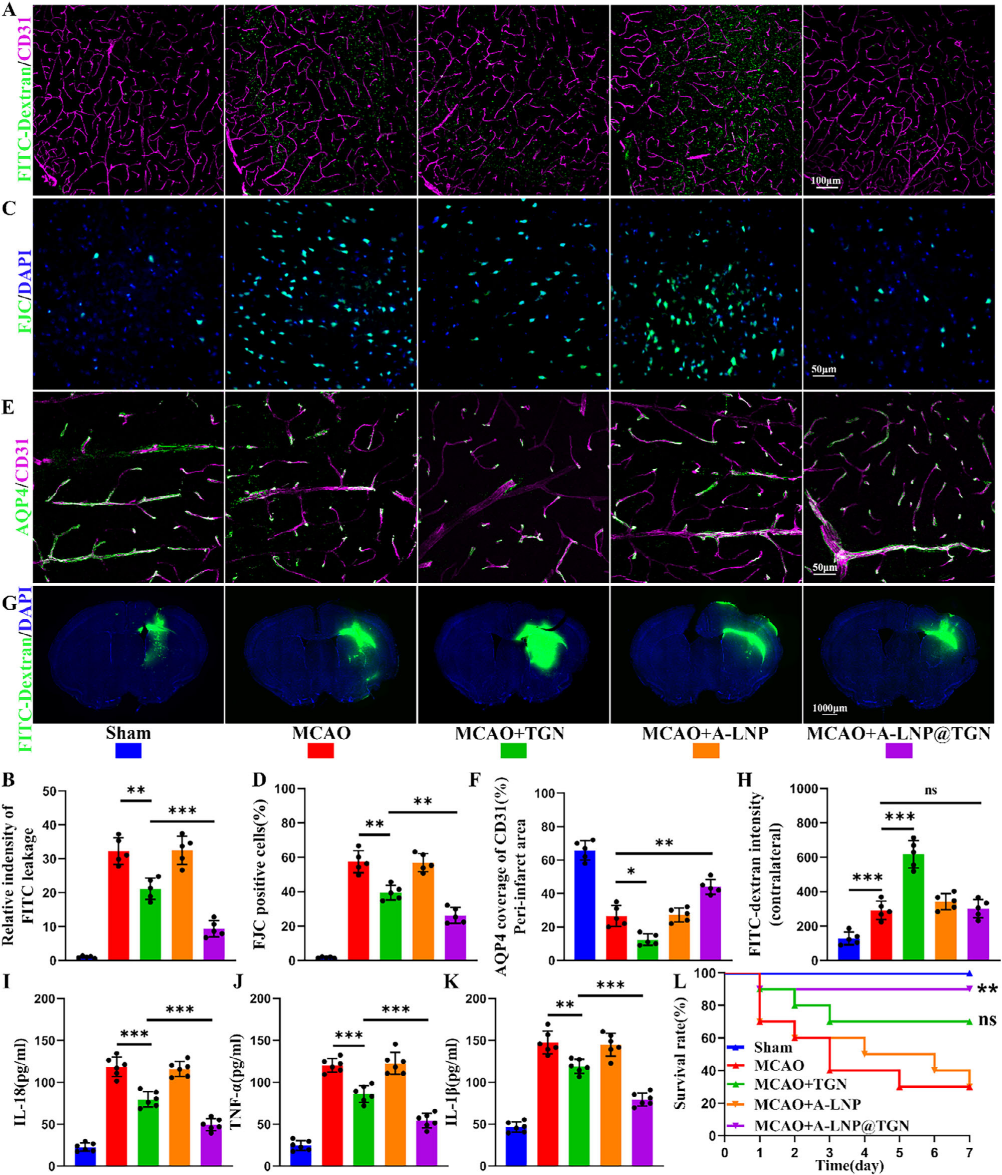
A‐LNP@TGN mitigates MCAO‐induced brain injury while preserving glymphatic clearance function. A) Representative immunofluorescence images of BBB permeability in different groups. B) Statistical result of BBB permeability test (*n* = 5). C) Representative FJC staining images. D) Statistical result of FJC staining (*n* = 5). E) Representative immunofluorescence images of AQP4‐CD31 colocalization to assess polarization of AQP4. F) Statistical result of AQP4‐CD31 colocalization (*n* = 5). G,H) Representative immunofluorescence images of glymphatic clearance and statistical results. I–K) Enzyme‐linked immunosorbent assay (ELISA) results of IL‐1β, IL‐18, and TNF‐α for each group (*n* = 6). L) Survival rate of each group (*n* = 10, vs MCAO). Data represent the mean ± SD, ns = no significant, ^*^ *p* < 0.05, ^**^p < 0.01, ^***^ *p* < 0.001.

Subsequent BBB permeability experiments revealed the greatest reduction in FITC vascular extravasation in the A‐LNP@TGN group compared to other groups after MCAO surgery, indicating better preservation of BBB integrity (**Figure** **7**A,B). Correspondingly, western blot results confirmed that TGN reversed the downregulation of tight junction protein occludin post‐MCAO, and A‐LNP@TGN exhibited a better effect (Figure S14, Supporting Information). Besides, FJC staining showed substantially fewer degenerating neurons in A‐LNP@TGN‐treated mice compared to other groups following MCAO surgery (Figure 7C,D), further validating its superior neuroprotection. Then, we detected perivascular AQP4 coverage in peri‐infarct regions to evaluate the effect of A‐LNP@TGN on the glymphatic system according to a previous report.^[^ ^23^ ^]^ While TGN decreased AQP4‐CD31 colocalization vs the MCAO group, A‐LNP@TGN treatment significantly reversed this unfavorable effect (Figure 7E,F). FITC clearance assays further demonstrated that compared with the MCAO group, TGN significantly delayed FITC clearance in the contralateral cerebral cortex of mice with ischemic stroke, whereas the A‐LNP@TGN group showed no significant difference (Figure 7G,H). Western blot confirmed that AQP4 expression was significantly upregulated in the ipsilateral hemisphere after ischemic stroke, while remaining largely unchanged in the contralateral hemisphere. In contrast to TGN, which suppressed AQP4 expression in both cerebral hemispheres, A‐LNP@TGN treatment selectively normalized the pathologically elevated AQP4 levels in the injured region while maintaining physiological expression in the unaffected area (Figure S15, Supporting Information). These findings suggested that TGN substantially impairs glymphatic clearance function after ischemic stroke, while A‐LNP@TGN mitigates this adverse effect. In addition, the nanoparticle formulation also amplified TGN's inherent anti‐inflammatory properties,^[^ ^33^ ^]^ demonstrating more pronounced reductions in pro‐inflammatory cytokines such as IL‐1β, IL‐18, and TNF‐α compared to TGN alone (Figure 7I–K). These multifaceted therapeutic benefits of A‐LNP@TGN collectively translated to significantly improved survival outcomes and weight recovery following MCAO (Figure 7L) (Figure S16, Supporting Information). Moreover, H&E staining demonstrated no pathological changes in major organs after receiving A‐LNP@TGN treatment in the ischemic stroke model (Figure S17 Supporting Information). Together, these findings demonstrated that LNP‐mediated TGN delivery not only enhanced therapeutic efficacy across multiple pathological parameters but also reduced off‐target TGN's adverse effects on whole brain glymphatic system function.

### 2.7 A‐LNP@TGN Attenuates Stroke‐Driven Pathogenic Signaling Alterations

**Figure 8:**
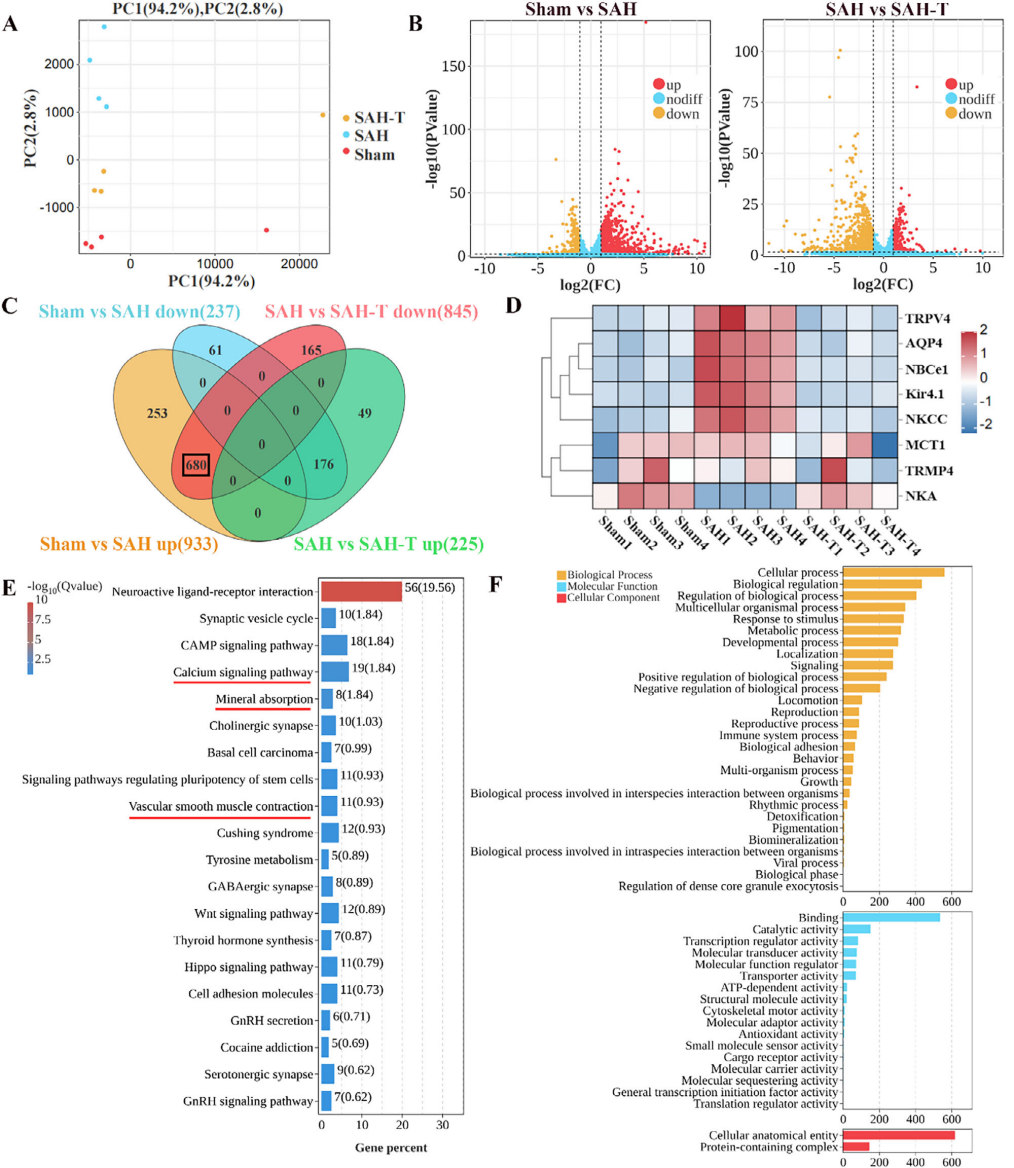
A‐LNP@TGN attenuates SAH‐driven pathogenic signaling alterations. A) PCA analysis of Sham, SAH, and SAH‐T samples (SAH‐T = SAH+A‐LNP@TGN, *n* = 4). B) Volcano plot of DEGs across different groups. C) Venn diagram of DEGs across different groups. D) Heatmap of changes of brain edema‐associated DEGs across different groups. E) KEGG of 680 interested DEGs. F) GO of 680 interested DEGs.

To provide multiscale insights into stroke‐induced injury after A‐LNP@TGN treatment. In terms of SAH, RNA‐seq analysis of brain tissues from Sham, SAH, and A‐LNP@TGN‐treated SAH mice (SAH‐T) was conducted. Principal component analysis (PCA) revealed closer transcriptional clustering between Sham and A‐LNP@TGN‐treated groups than the SAH group, suggesting partial restoration of injury‐induced gene expression profiles (**Figure** **8**A). While two samples from each of the Sham and A‐LNP@TGN groups showed minor deviations, inter‐group consistency remained robust (Figure S18, Supporting Information). Volcano plots highlighted differentially expressed genes (DEGs) across groups (Figure 8B). Meanwhile, the heatmap revealed DEGs in response to SAH compared to Sham and SAH with A‐LNP@TGN treatment compared to SAH. Hierarchical clustering identified 50 representative DEGs. The Kyoto Encyclopedia of Genes and Genomes (KEGG) pathway analysis and Gene Ontology (GO) analysis systematically mapped pathway enrichment and functional annotation associated with DEGs from Sham vs SAH and SAH vs SAH‐T (Figure S19–S21, Supporting Information). Intersection analysis identified 680 genes upregulated post‐SAH but downregulated after A‐LNP@TGN administration (Figure 8C). In addition, cerebral edema‐related genes were selected especially,^[^ ^34^, ^35^ ^]^ heatmap demonstrated SAH‐induced upregulation of TRPV4, AQP4, NBCe1, Kir4.1, and NKCC, alongside downregulated ATPase expression. Notably, A‐LNP@TGN treatment effectively reversed these gene alterations (Figure 8D). Furthermore, KEGG analysis and GO analysis further revealed that A‐LNP@TGN mediated‐680 DEGs were implicated in key pathways of brain edema and hypoperfusion, such as the calcium signaling pathway, mineral absorption, and vascular smooth muscle contraction (Figure 8E,F). These findings suggested that A‐LNP@TGN exerted multiscale therapeutic effects to improve post‐SAH brain injury.

**Figure 9:**
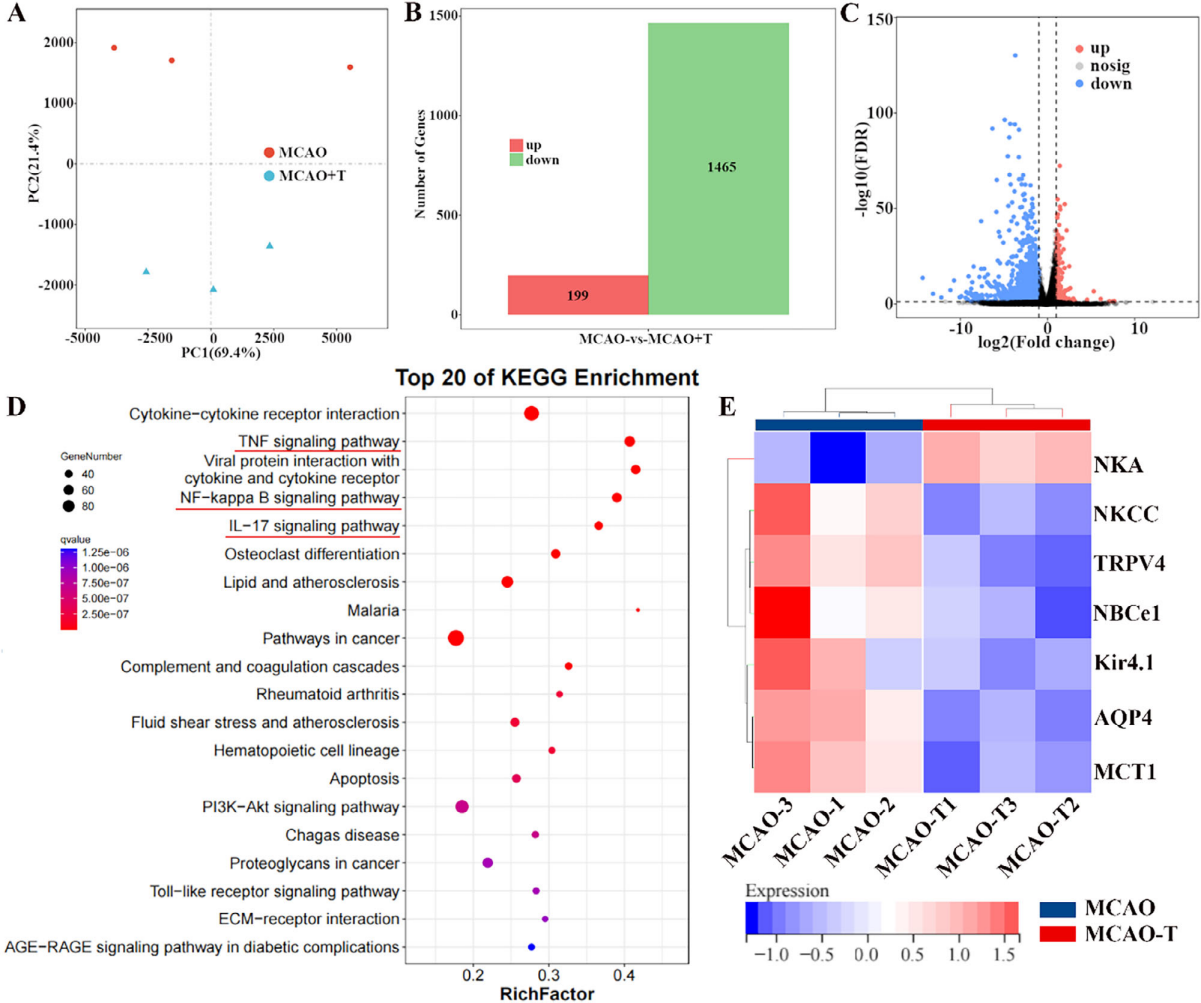
A‐LNP@TGN attenuates MCAO‐induced pathological signaling alterations. A) PCA analysis of MCAO, MCAO+A‐LNP@TGN samples (MCAO‐T = MCAO+A‐LNP@TGN, *n* = 3). B) Summary of up‐ and down‐regulated DEGs before and after A‐LNP@TGN treatment in the MCAO model. C) Volcano plot of DEGs across different groups. D) KEGG of DEGs in response to MCAO receiving A‐LNP@TGN. E) Heatmap of changes in brain edema‐associated DEGs across different groups.

In parallel, RNA‐seq analysis was conducted in the MCAO model of ischemic stroke, comparing brain tissues before and after A‐LNP@TGN treatment. PCA revealed clear segregation between the MCAO and A‐LNP@TGN‐treated groups (**Figure** **9**A), with strong intra‐group reproducibility (Figure S22, Supporting Information). Treatment with A‐LNP@TGN resulted in 199 upregulated and 1465 downregulated DEGs relative to MCAO controls (Figure 9B), as visualized by a volcano plot (Figure 9C) and a heatmap (Figure S23, Supporting Information). KEGG pathway analysis indicated that the benefits of A‐LNP@TGN were associated with the significant modulation of key inflammatory and apoptotic pathways, including TNF, NF‐κB, and IL‐17 signaling (Figure 9D). Furthermore, the treatment markedly suppressed the expression of cerebral edema‐associated genes (Figure 9E). These transcriptomic findings were corroborated by western blot analysis, which confirmed the downregulation of AQP4 protein and attenuation of key inflammatory mediators.

## 3 Discussion

Our study bridged an important knowledge gap regarding the different effects of TGN in hemorrhagic and ischemic stroke. While both subarachnoid hemorrhage (SAH) and ischemic stroke share common pathological features of cerebral edema and microcirculatory hypoperfusion,^[^ ^7^, ^8^, ^9^ ^]^ and the protective effects of TGN treatment against ischemic stroke are well‐established,^[^ ^25^, ^26^, ^33^ ^]^ our investigation revealed a distinct therapeutic efficacy of TGN in SAH. TGN effectively reduced post‐SAH brain edema and improved microcirculation disturbance through AQP4 suppression, but it simultaneously exacerbated glymphatic clearance dysfunction and impaired neurotoxin elimination, thereby aggravating neuroinflammation and neuronal damage. In ischemic stroke, ischemia‐mediated hypoxia is the primary cause of subsequent pathological damage, whereas in hemorrhagic stroke, secondary brain injury is mostly triggered by blood‐derived neurotoxic components infiltrating brain tissue.^[^ ^36^ ^]^ Systematic AQP4 inhibition may impair glymphatic clearance of these toxins following SAH, exacerbating brain injury risk—potentially explaining the differential therapeutic effects of TGN across stroke subtypes. These observations aligned with recent research on TGN's roles in an intracerebral hemorrhage model.^[^ ^28^ ^]^ This created a critical therapeutic dilemma where the benefits of TGN in suppressing edema were overshadowed by its global impairment of glymphatic function. To address this challenge, we developed a targeted LNP delivery system incorporating angiopep‐2 peptide that achieved region‐specific TGN delivery to injury sites while preserving whole‐brain glymphatic function. Both in vitro and in vivo studies confirmed the uptake of A‐LNP@TGN by astrocytes without significant off‐target toxicity. Most importantly, this targeted approach maintained the therapeutic advantages of TGN in mitigating brain edema and hypoperfusion while avoiding its detrimental effects on glymphatic clearance, as further corroborated by RNA sequencing analyses. These findings represent a significant advancement in hemorrhagic stroke therapeutics by overcoming previous limitations of systemic AQP4 modulation.

Furthermore, our study provided novel insights into the therapeutic potential of AQP4 modulation for addressing post‐ischemic no‐reflow, a microcirculatory dysfunction affecting up to 25% of ischemic stroke patients despite successful recanalization, which significantly compromises clinical outcomes.^[^ ^37^ ^]^ Previous studies established TGN's ability to reduce cerebral edema and neuroinflammation through AQP4 suppression, thereby decreasing infarct volume and neurological deficits following cerebral ischemia. Building upon established knowledge that astrocytic endfeet swelling contributes to microcirculatory disturbance post‐ischemic stroke,^[^ ^38^ ^]^ we found first that TGN effectively ameliorated ischemic stroke‐induced no‐reflow phenomenon. The resulting blood flow restoration attenuated key pathological progress, including blood‐brain barrier disruption, neuronal injury, and neuroinflammatory responses. Moreover, the LNP‐based targeted delivery system significantly potentiated these beneficial effects of TGN. Importantly, unlike systemic TGN administration, A‐LNP@TGN preserved glymphatic clearance function in peri‐ischemic regions, thereby enhancing therapeutic efficacy. The RNA‐seq results indicated that A‐LNP@TGN ameliorates brain injury after stroke via multi‐target mechanisms. Previous studies have shown that AQP4 functionally synergizes with TRPV4, which is implicated in the pathogenesis of cerebral edema.^[^ ^11^ ^]^ Inhibition of AQP4 expression has been shown to improve microcirculatory disturbances in cerebral edema models.^[^ ^15^ ^]^ Additionally, TGN has been reported to attenuate inflammatory responses and neuronal apoptosis following ischemic stroke.^[^ ^33^ ^]^ Consistent with these findings, our RNA‐seq analysis revealed that A‐LNP@TGN downregulated genes associated with post‐stroke cerebral edema and modulated key pathways involved in microcirculatory impairment, neuroinflammation, and apoptosis.

Several important considerations emerge from these findings. Biodistribution studies confirmed that A‐LNP accumulates not only in the brain but also in the liver. Although no significant hepatic metabolic or histopathological alterations were observed in our toxicity assessments, further optimization of brain targeting efficiency is warranted to minimize off‐target accumulation. In this context, nasal delivery systems represent a promising alternative strategy. By leveraging the olfactory bulb pathway, intranasal administration could enhance brain‐specific delivery while bypassing systemic circulation and reducing hepatic first‐pass effects.^[^ ^39^ ^]^ Prior work suggests the expression of AQP4 is critical for glymphatic clearance function, while the depolarization of AQP4 seems to affect the clearance of large instead of small molecules.^[^ ^40^ ^]^ This might explain why the SAH group receiving TGN treatment exhibited worse glymphatic clearance dysfunction than other groups. Although our study provided preliminary validation of A‐LNP@TGN's ability to preserve whole‐brain glymphatic function post‐stroke, a more comprehensive characterization of its effects on intracranial clearance dynamics warrants further investigation. Additionally, the incomplete perfusion restoration after A‐LNP@TGN treatment suggested other underlying no‐reflow mechanisms like neutrophil capillary plugging still exist.^[^ ^41^, ^42^ ^]^ Future studies should verify the effect of combining A‐LNP@TGN with leukocyte‐targeting therapies on stroke‐induced microcirculatory hypoperfusion. In summary, these findings demonstrated inhibition of AQP4 could serve as a therapeutic target for post‐stroke no‐reflow, while the engineered LNP platform potentiated TGN's neuroprotection against ischemic brain injury. This work provides a promising new avenue for the treatment of ischemic cerebral injury and other hypoperfusion‐related neurovascular disorders.

## 4 Conclusion

Collectively, this study demonstrates that while TGN exerts dual benefits in alleviating post‐SAH cerebral edema and microcirculatory dysfunction, its clinical translation is limited by off‐target glymphatic suppression. To overcome this limitation, we developed an angiopep‐2‐functionalized LNP system that achieved lesion‐specific delivery and spatiotemporally controlled release of TGN. This system selectively suppressed AQP4 overexpression in the injured hemisphere without compromising its physiological levels in healthy tissue, thereby preserving global glymphatic function. Experimental validation demonstrates that A‐LNP@TGN not only potentiates suppression of brain edema and recovery of cerebral perfusion but also preserves the neurotoxic clearance capacity of the glymphatic system. Notably, the platform's therapeutic universality is further confirmed in ischemic stroke, where TGN‐mediated no‐reflow improvement synergizes with glymphatic preservation to enhance neuroprotection. These findings advance our understanding of AQP4‐targeted therapies and offer a precision nanomedicine strategy for stroke therapy.

## 5 Experimental Section

### Preparation and Characterization of A‐LNP@TGN

A 200 µg portion of TGN was incorporated into a 100 µL ethanolic solution containing a lipid mixture of Dlin‐MC3‐DMA, DSPC, cholesterol, DMG‐PEG2000‐Angiopep‐2 (Sangon Biotech, China), combined at a molar ratio of 50:10:38.5:1.5. This ethanolic lipid solution was subsequently added to 300 µL of sodium citrate buffer (pH 4.0). Dialysis against phosphate‐buffered saline (PBS, pH 7.4) was performed for 12 h at 4 °C to remove ethanol and unencapsulated components, yielding LNP@TGN, A‐LNP@TGN, A‐LNP without TGN were prepared using the same concentrations. The brainless targeted functional LNP (vehicle) was prepared by substituting DMG‐PEG2000 for DMG‐PEG2000‐angiopep‐2 with equal amounts of DMG‐PEG2000. In addition, DiR‐labeled samples were prepared by adding 0.2 µg DiR to ethanol in the same manner.

The size and surface Zeta potential of samples were characterized using Zetasizer Nano ZS (Malvern, UK). The A‐LNP@TGN was dropped on a copper grid and stained with phosphotungstic acid, dried, and then the morphology was observed by TEM (JEOL, Japan). Multiple centrifuge tubes containing 10 mL of PBS buffer (pH 7.4) were prepared, and a 3000 D dialysis bag containing 1 mL of A‐LNP@TGN was added to each centrifuge tube. The samples were placed on a thermostatic shaker at 37 °C (200 rpm). The solution in the dialysis bag was collected at various times, the membrane was broken using Triton‐100, and the TGN release behavior was measured by HPLC (ThermoFisher Scientific, USA).

### Cell Culture

Primary astrocytes were isolated from postnatal mice (<24 h) using standardized protocols.^[^ ^43^ ^]^ Briefly, cortical tissues were dissected and meticulously cleared of meningeal layers. Tissue dissociation was carried out with 2.5% trypsin (Gibco, Grand Island, NY, USA), which was subsequently neutralized with a complete culture medium (DMEM supplemented with 10% fetal bovine serum (FBS; Gibco) and penicillin‐streptomycin (Gibco)). Following centrifugation (300 x g, 5 min), the cell pellet was resuspended in fresh medium and maintained in a humidified incubator (37 °C, 5% CO_2_). Astrocyte purity was verified by GFAP immunostaining. To establish an in vitro model of hemorrhagic stroke, cultured astrocytes were exposed to 50 µM hemoglobin (Sigma) for 12 h. The bEnd.3 cells were purchased from Pricella (China). They were cultured in a complete culture medium and maintained in a humidified incubator (37 °C, 5% CO_2_).

### Cellular Uptake and Effect of A‐LNP@TGN on Astrocytes

Primary astrocytes were seeded in a 24‐well plate and incubated at 37 °C overnight. To evaluate the cellular uptake of A‐LNP@TGN in astrocytes under subarachnoid hemorrhage (SAH) conditions, cells were treated with hemoglobin and DiR‐labeled A‐LNP@TGN for 6 h. After incubation, the cells were washed and fixed, followed by overnight incubation at 4 °C with mouse anti‐GFAP antibody (1:200, Servicebio). The next day, cells were washed with PBS and incubated for 1 h at room temperature with a secondary antibody (AlexaFluor‐488‐conjugated donkey anti‐mouse IgG, 1:500; Invitrogen). After additional washes, nuclei were counterstained with 4′,6‐diamidino‐2‐phenylindole (DAPI) for 10 min at room temperature. Fluorescent images were acquired using a confocal microscope (Nikon, Japan). Additionally, immunofluorescence staining was performed to evaluate whether A‐LNP@TGN attenuated hemoglobin‐induced astrocyte swelling.

### Flow Cytometry

Primary astrocytes were seeded in a 24‐well plate and incubated at 37 °C overnight. Following a 24‐h incubation period, these cells were treated with 1 mL of the sample medium at a final concentration of 500 µg Ml^−1^ according to different groups, followed by an additional 6‐h incubation. After the culture period, the cells were washed, harvested, and resuspended in 200 µL of PBS buffer supplemented with 1% BSA. Cell analysis was conducted using a flow cytometer (Beckman, USA), and the acquired data were analyzed using FlowJo software.

### Cell Toxicity Examination

Cell toxicity examination was assessed using the CCK‐8 (Dojindo, Japan). Primary astrocytes were seeded in 96‐well plates and cultured for 24 h at 37 °C. These cells were then treated with different concentrations of A‐LNP@TGN, 6 parallel wells per concentration. The cytotoxicity was measured according to the CCK‐8 kit instructions.

### Detection of A‐LNP@TGN to Cross BBB

The bEnd.3 cells were cultured in Transwell. When the trans‐endothelial electrical resistance was greater than 200 Ω, BBB formation in vitro was successful. Primary astrocytes were cultured in the lower compartment of the culture plate. After preparing the astrocytes SAH model, DiR‐labeled LNP@TGN or A‐LNP@TGN were added to the upper chamber of Transwell for different groups. After 6 h of culture, the astrocytes in the lower chamber were washed and fixed, followed by incubating with DAPI for 10 min to measure fluorescence intensity.

### Hemolytic Test

Mouse blood was collected using an anticoagulant tube, an equal volume of PBS was added, and gently mixed at 3000 rpm for 10 min. After washing the red blood cells twice with PBS, resuspend the red blood cells with PBS to prepare a 4% red blood cell suspension. The nanoparticles were mixed with red blood cell suspension in equal volumes to obtain 2% red blood cell suspension. The negative control group was mixed with red blood cell suspension and an equal volume PBS, and the positive control group was mixed with red blood cell suspension and an equal volume of deionized water. Mix the mixtures gently and incubate for 1 h at 37 °C and a shaker at 180 rpm. At the end of the incubation, the mixture was centrifuged at 3000 rpm for 10 min to collect the supernatant. Pipette 100 µL of supernatant into a 96‐well plate and measure absorbance at 540 nm using a multimode microplate reader. Hemolysis rate = [(ODexperimental group – ODnegative control group) / (ODpositive control group – ODnegative control group)] × 100%.

### Pharmacokinetic Analysis

The in vivo pharmacokinetic evaluation of DiR‐labeled A‐LNP@TGN was performed in a mouse model. The formulation was administered intravenously at a volume of 200 µL via the tail vein. Blood samples (50 µL each) were obtained from the same site before dosing and at designated intervals thereafter. Immediately after collection, each sample was mixed with an equal volume of PBS. Fluorescence emission at 778 nm was quantified using a microplate reader. Based on the resulting concentration–time profile, essential pharmacokinetic metrics—such as elimination half‐life (T_1_/_2_), area under the curve (AUC), and systemic clearance (CL) were derived.

### Animal

Adult male C57BL/6 mice (6‐8 weeks old, weighing 20–25 g) were obtained from the Animal Experiment Center of Southern Medical University (Guangzhou, China). All experimental procedures were performed in compliance with the guidelines established by the Institute of Laboratory Animal Resources. The study protocol was approved by the Ethics Committee of Zhujiang Hospital, Southern Medical University (Approval No. LAEC‐2024‐284). Mice were maintained under standard housing conditions at the Zhujiang Hospital Animal Experiment Center with a 12‐h light/dark cycle and provided with food and water ad libitum.

### Establishment of Stroke Models

The SAH model was established using endovascular perforation following established protocols.^[^ ^44^ ^]^ Briefly, C57BL/6 mice were anesthetized and surgically prepared to expose the common carotid artery (CCA) and its branches. After ligating and transecting the external carotid artery (ECA), a sharpened nylon suture was introduced through the ECA into the CCA. The suture was carefully advanced along the internal carotid artery (ICA) until resistance indicated perforation at the bifurcation of the anterior and middle cerebral arteries. Immediate suture withdrawal followed perforation, with subsequent wound closure. The mice in the sham group accepted the same procedure, except for vascular perforation. A standardized six‐segment grading system of the basal cistern was employed to ensure consistent hemorrhage severity.^[^ ^45^ ^]^ Total SAH scores were calculated by summing individual segment scores. Animals scoring below 8 were excluded from subsequent analyses.

The transient middle cerebral artery occlusion model was generated as previously described,^[^ ^46^ ^]^ with modifications from the SAH procedure: (1) A silica‐coated nylon monofilament replaced the sharpened suture; (2) The filament was advanced to occlude the MCA origin without vessel perforation. After 60 min of occlusion, the filament was withdrawn to permit reperfusion. Mice in the sham‐operated group received the same procedures mentioned above except for occlusion. TGN‐020 was administered intraperitoneally immediately post‐surgery, while the LNP‐based TGN delivery system was administered via tail vein injection immediately after model induction.

### In Vivo Imaging System

Upon successful induction of the SAH model, three experimental groups received intravenous tail vein injections: PBS (control), non‐targeted DIR‐labeled LNP@TGN, and DIR‐labeled A‐LNP@TGN (including 100 ug TGN, 200ul). After 8 h, animals were sacrificed for organ collection, and DIR fluorescence distribution was quantified using a small animal live imager (PerkinElmer).

### Brain Edema Analysis

Brain water content measurement was applied to quantitatively assess the degree of cerebral edema. In the SAH model groups, animals were sacrificed at three time points following SAH (1, 3, and 5 days). Brains were promptly harvested and systematically dissected into four distinct regions: bilateral cortical hemispheres, brainstem, and cerebellum. Each tissue sample was immediately weighed to obtain wet weight, followed by dehydration at 95 °C for 24 h to determine dry weight. The percentage of brain water content was calculated using the following formula: Brain water content = (wet weight – dry weight)/wet weight × 100%. For the MCAO model assessment, animals were sacrificed 24 h post‐occlusion. Since ischemic stroke produces more severe cerebral edema than hemorrhagic stroke, the collected brains were divided into three anatomical sections. Only ipsilateral (left) hemispheric samples were selected for further analysis, as this region exhibits the most significant pathological changes following ischemic stroke.

### TEM

Briefly, mice were euthanized and transcardially perfused with 4% glutaraldehyde for primary fixation. The sensory cortices were then carefully dissected and fixed further in 2% osmium tetroxide in sodium cacodylate buffer. Following fixation, the samples were stained with 2% uranyl acetate, dehydrated in a series of graded ethanol solutions, and finally embedded in Araldite resin for ultrathin sectioning. Ultrathin sections were prepared using an Ultramicrotome (Leica Microsystems) and subsequently stained with uranyl acetate and lead citrate for contrast enhancement. For quantitative assessment, randomly capture representative images from fields exhibiting complete vascular cross‐sections accompanied by astrocytic endfeet. Capture and analysis of images were performed by investigators who were unaware of treatment allocation.

### Immunofluorescent Staining

Briefly, at 24 h post‐stroke induction, anesthetized mice underwent transcardial perfusion with 50 mL phosphate‐buffered saline (PBS) followed by 50 mL paraformaldehyde (PFA). Brains were rapidly extracted and post‐fixed in PFA for 24 h at 4 °C before being dehydrated through a sucrose gradient, embedded in optimal cutting temperature compound, and sectioned into 10 µm thick slices using a cryostat. After PBS washing, sections were blocked with 5% donkey serum for 1 h at room temperature, then incubated overnight at 4 °C with primary antibodies: mouse anti‐APQ4 (1:200, Servicebio); Goat anti‐CD31 (1:200, Servicebio). The next day, sections were washed and incubated for 1 h at room temperature with Alexa Fluor 647‐conjugated donkey anti‐goat IgG (1:500) and Alexa Fluor 488‐conjugated donkey anti‐mouse IgG (1:500) secondary antibodies, followed by DAPI nuclear counterstaining for 10 min. Fluorescence images were acquired using a confocal microscope. To evaluate A‐LNP@TGN's effects on the post‐MCAO glymphatic system, AQP4 coverage in peri‐infarct regions from the ipsilateral cortical of the specific coronal layer was analyzed per brain by investigators blinded to treatment groups.

### FJC Staining

FJC staining was conducted on frozen brain sections to detect degenerating neurons following standard immunofluorescence protocols, with all procedures strictly adhering to the manufacturer's guidelines (Biosensis, Australian). The fluorescent images were captured using an epifluorescence microscope, and a quantitative assessment of neuronal degeneration was performed by a blinded investigator, with results expressed as the percentage of FJC‐positive cells relative to DAPI‐positive cells for accurate analysis.

### Cellular Uptake of A‐LNP@TGN by Astrocyte

Following SAH induction, mice received immediate intravenous administration of DiR‐labeled A‐LNP@TGN via tail vein injection. Frozen sections were prepared according to the description of immunofluorescence staining. Astrocytes were specifically labeled using mouse anti‐GFAP primary antibody (1:200 dilution, Servicebio) for immunofluorescence analysis. A confocal fluorescence microscope was then employed to examine the spatial colocalization patterns between GFAP‐positive astrocytes and DiR fluorescence signals within the injury regions.

### Evaluation of Microcirculation Perfusion

Experimental mice were anesthetized 24 h after stroke induction and intravenously administered FITC‐conjugated dextran (70 kDa, 25 mg mL^−1^ in physiological saline). After allowing 1 h for systemic circulation, brains were carefully harvested and immersion‐fixed in PFA at 4 °C for five days. The fixed tissues underwent sucrose gradient cryoprotection followed by preparation of 70‐µm‐thick coronal sections using a cryostat. Microvascular networks were visualized through immunostaining with goat anti‐CD31 antibody (1:100, Servicebio), while perfused vasculature was identified by FITC‐dextran distribution. Confocal fluorescence microscopy with z‐stack acquisition (2 µm intervals × 20 steps) and subsequent 3D reconstruction was employed to evaluate perfusion changes in the injury regions. Quantitative assessment of microcirculatory perfusion was performed by calculating the ratio of the FITC‐positive (perfused) area to the total CD31‐positive (vascular) area, with all imaging procedures and analyses conducted by investigators blinded to treatment groups to ensure objective evaluation.

### Evaluation of BBB Permeability

To assess BBB disruption after MCAO‐induction, mice received an intravenous injection of FITC‐dextran (70 kDa) 24 h post‐surgery. Following a 1‐h systemic circulation period, the animals were transcardially perfused with PBS, followed by PFA fixation to preserve vascular leakage. Brain tissues were then cryosectioned into 70‐µm‐thick frozen slices using the aforementioned immunofluorescence protocols. For microvascular visualization, sections were stained with a goat anti‐CD31 antibody (1:100, Servicebio). BBB disruption was evaluated by measuring perivascular FITC‐dextran extravasation using confocal microscopy, with signal intensity quantified to determine leakage severity. To ensure unbiased assessment, all imaging and data analyses were conducted by investigators blinded to treatment groups.

### Western Blot

Protein samples were separately extracted from the ipsilateral and contralateral cerebral cortices of stroke model mice using RIPA lysis buffer (Cwbio, China). Equal protein amounts were separated by 10% SDS‐PAGE and transferred to PVDF membranes. After 3‐h blocking with skim milk, membranes were incubated overnight at 4 °C with primary antibodies against AQP4 (1:1000, Proteintech), IL‐18 (1:1000, CST), IL‐1β (1:1000, CST), TNF‐α (1:1000, Proteintech), and Occludin (1:1000, CST), using β‐actin as a loading control. Following 1‐h incubation with HRP‐conjugated secondary antibody at room temperature, protein bands were detected using an ECL system (WBKLS0100, Millipore) and quantified with ImageJ software.

### Stereotactic Injection

Stereotactic injection was used to assess the glymphatic system clearance function according to previous literature.^[^ ^47^ ^]^ 6 h post‐SAH surgery, mice from different groups were anesthetized and positioned in a stereotaxic frame. Following the scalp incision, a burr hole was drilled using a micro‐drill (2 mm lateral and 2 mm posterior to bregma). A stereotaxic needle was inserted 4.5 mm below the skull surface, and 2 µL of FITC‐dextran (70 kDa) was infused at 0.2 µL min^−1^ using a microinjection pump. The needle remained in situ for 10 min post‐injection to prevent reflux before wound closure. 2 h later, mice underwent transcardial perfusion with PBS followed by PFA. After brain extraction and fixation, 70 µm‐thick frozen sections were prepared as described before. Brain slices were stained for nuclei. Then, fluorescence microscopy was used to image the brain sections. ImageJ software was employed for quantitative analysis of the overall whole‐brain fluorescent signal intensity of specific coronal layers. The injection coordinates for the ischemic stroke model were adjusted (to 2 mm lateral and 0.5 mm anterior to bregma, 3.25 mm below the skull surface), while all other procedures remained unchanged.

### Evaluation of Neurological Deficit

Neurological deficits were evaluated using standardized behavioral tests. For SAH‐induced neurological impairments, the modified Garcia test was employed as previously described,^[^ ^44^ ^]^ assessing six sensorimotor functions (spontaneous activity, limb movement, forelimb outstretching, climbing, trunk touch, and vibrissal response) with individual scores of 0–3 (total score range: 0–18, where higher scores indicate milder deficits). MCAO‐induced neurobehavioral deficits were measured by mNSS. The mNSS ranges from 0 (normal) to 18 (most severe) for movement, sensation, balance, and reflexes.^[^ ^48^ ^]^ Rotarod test was performed to assess changes in balance and motor coordination post‐SAH and post‐MCAO. The mice underwent a 2‐day training prior to operation, which gradually accelerated from 4 to 15 rpm in 5 min. The mean time of latency to fall was measured when the mice fell off the rod in three trials.

### Section Staining

At 24 h post‐SAH, deeply anesthetized mice underwent transcardial perfusion with 50 ml PBS followed by 50 ml PFA. Brains were rapidly extracted and post‐fixed in PFA for 24 h at 4 °C. Following paraffin embedding, tissues were sectioned at 5 µm thickness and processed for H&E and Perls Prussian blue staining using standardized protocols. After sequential dehydration in absolute alcohol and xylene clearing, sections were mounted with neutral resin for microscopic examination. H&E staining enabled the evaluation of edema‐related morphological alterations, while Perls staining quantified iron deposition. All histological analyses were performed by a blinded investigator using a light microscope (Leica, Germany).

### Evaluation of Cerebral Blood Flow and Brain Infarction

Regional cerebral blood flow in the stroke‐affected area was monitored using a laser Doppler perfusion imager (RFSLI ZW, RWD) at predetermined time points (1 h pre‐ischemia, 1 h ischemia, and 24 h post‐MCAO). During imaging, a saline solution was applied to maintain cranial window moisture for optimal imaging quality. Standard acquisition parameters were maintained (gain: 200; exposure: 20 ms; frame rate: 1.00 fps; background threshold: 10; working distance: 190 mm). Following cerebral blood flow assessment, mice were euthanized for TTC staining. Brains were carefully extracted, briefly frozen at ‐20 °C for 30 min and sectioned coronally into 2 mm slices. These slices were incubated in 2% TTC solution (Solarbio, Beijing) at 37 °C for 15 min with periodic inversion, after which infarct volumes were quantified using ImageJ software.

### ELISA

At 24 h after successful MCAO modeling, the concentration of IL‐18, IL‐1β, and TNF‐𝛼 in mouse cerebral cortex homogenates was determined using an ELISA kit (MEIMIAN, China), strictly adhering to the manufacturer's protocol. Optical density values at a wavelength of 450 nm were recorded using a Tecan microplate reader (Tecan, Switzerland), facilitating the quantification of inflammatory cytokine levels.

### RNA‐Seq Analysis

At 24 h after SAH, cortical tissue samples located at the injury site were sent to Xiangyan Biotechnology Co. Ltd. (Guangzhou, China) for RNA‐seq library preparation. After clustering, transcriptome sequencing on the DNBSEQ‐A platform is performed, which generated the raw reads. Data quality was assessed by the FastQC tool after the removal of adaptor sequences, ambiguous “N” nucleotides (proportion of “N” > 5%), and low‐quality sequences (quality score <10). The ggplotR package was used to identify StringTie genes. The difference was the statistically significant threshold for |log2 fold change| ≥2 and P <0.05. GO enrichment, KEGG pathway analysis of genes and genomes, and heatmap and volcanic figure analysis were performed at https://www.omicshare.com (Gene Denovo, Guangzhou, China).

### Statistical Analysis

The independent two‐sample t‐test was used to compare two groups, while one‐way analysis of variance (ANOVA) followed by Tukey post hoc tests was used for comparison between multiple groups. All the statistical analysis was performed using GraphPad Prism 8 (GraphPad Software, USA), and the graphs were plotted. The statistical significance was indicated as ^*^ *p* < 0.05, ^**^ *p* < 0.01 and ^***^ *p* < 0.001. Data are shown as mean ± SD. The sample size in each experiment was specified in the figure legends.

### Ethics Approval Statement

All animal experiments were carried out in accordance with the Institute of Laboratory Animal Resources guidelines. Ethics approval was granted by the Ethics Committee of Zhujiang Hospital of Southern Medical University (approval number LAEC‐2024‐284).

## Conflict of Interest

The authors declare no conflict of interest.

## Supporting information



Supporting Information

## Data Availability

The data that support the findings of this study are available from the corresponding author upon reasonable request.
