# Supplementary material for: Amelioration of Post‐Stroke Edema and Microcirculatory Dysfunction via Targeted AQP4 Inhibition While Preserving the Glymphatic System
Source: Adv Sci (Weinh). 2025 Dec 12;13(10):e20118. doi: 10.1002/advs.202520118 (PMC12915113; doi:10.1002/advs.202520118)
Supplement: Supplementary file 1 — Supporting Information [file ADVS-13-e20118-s001.pdf]

Supporting Information

Amelioration of Post-Stroke Edema and Microcirculatory Dysfunction via Targeted AQP4 Inhibition while Preserving the Glymphatic System

Lei Jin, Zeyu Yang, Boyang Wei, Yu Wu, Longxiang Li, Jiaming Zhou, Xin Zhang, Fa Jin, Shixing Su, Yanchao Liu, Ran Li, Shenquan Guo, Xingwu Liu, Yu Cai, Hong Liu, Min Chen*, Wenchao Liu*, Chuanzhi Duan* and Xifeng Li*

Supplementary Figure Section

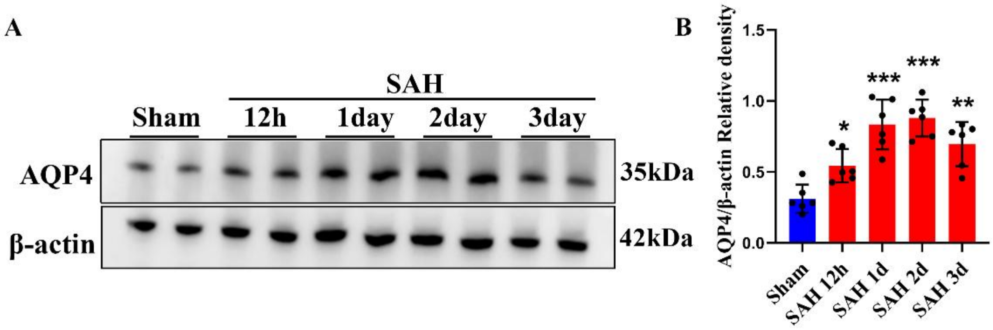

Figure S1. AQP4 expression after subarachnoid hemorrhage (SAH). A) Representative photographs of western blot. B) Corresponding statistical result (n = 6). Data are presented as the means ± SD, *P ＜ 0.05, **P ＜ 0.01, ***P ＜ 0.001 vs Sham group.

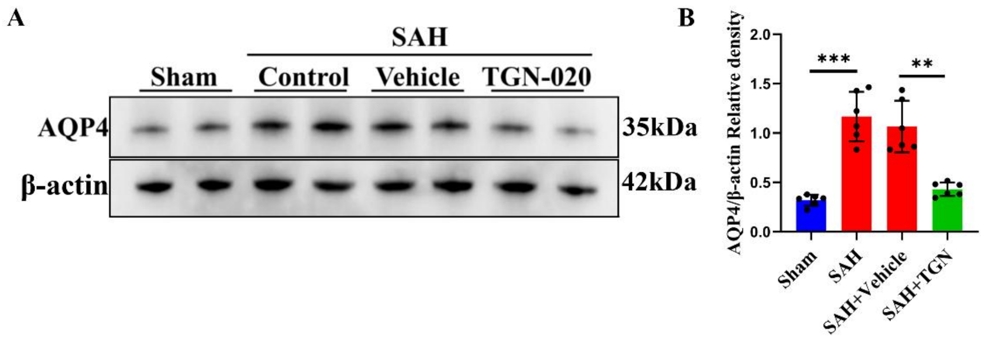

Figure S2. AQP4 expression in different groups. A) Representative photographs of western blot. B) Corresponding statistical result (n = 6). Data are presented as the means ± SD, $^{**}P < 0.01$, $^{***}P < 0.001$.

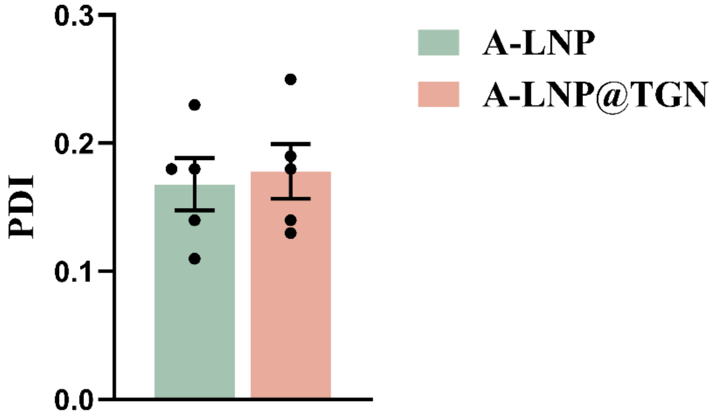

Figure S3. Polydispersity (PDI) of A-LNP and A-LNP@TGN (n = 5). Data are presented as the means ± SD.

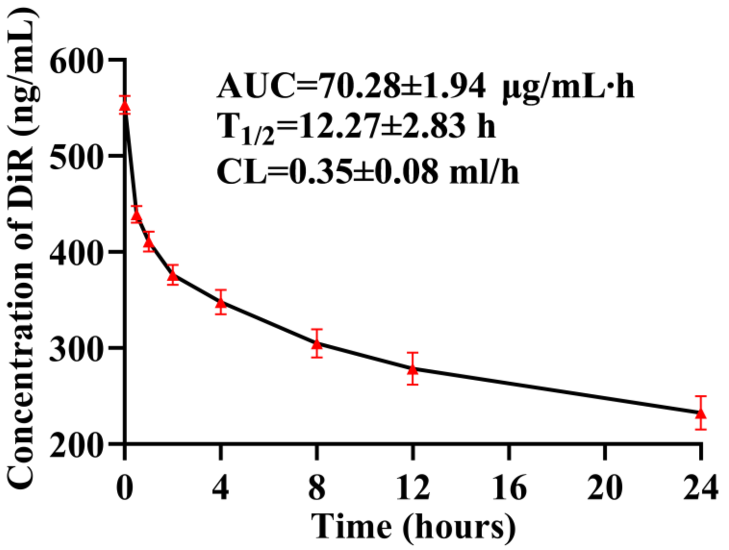

Figure S4. The concentration-time curve and pharmacokinetic parameters of A-LNP@TGN in the blood of mice after administration via the tail vein (n=5). Data were expressed as mean ± SEM.

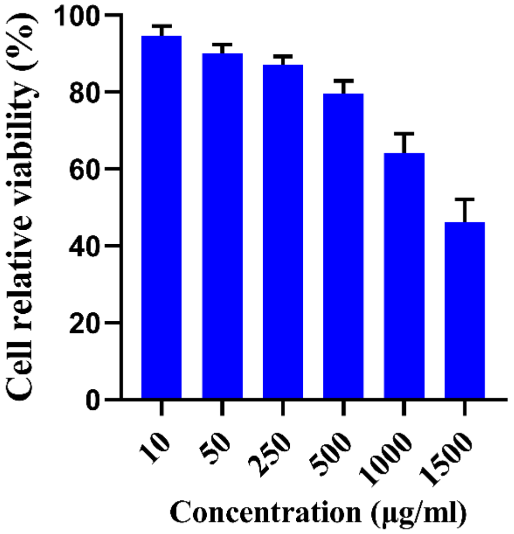

Figure S5. Biocompatibility of A-LNP@TGN evaluated by CCK-8 kit using primary astrocytes (n = 6). Data are presented as the means ± SD.

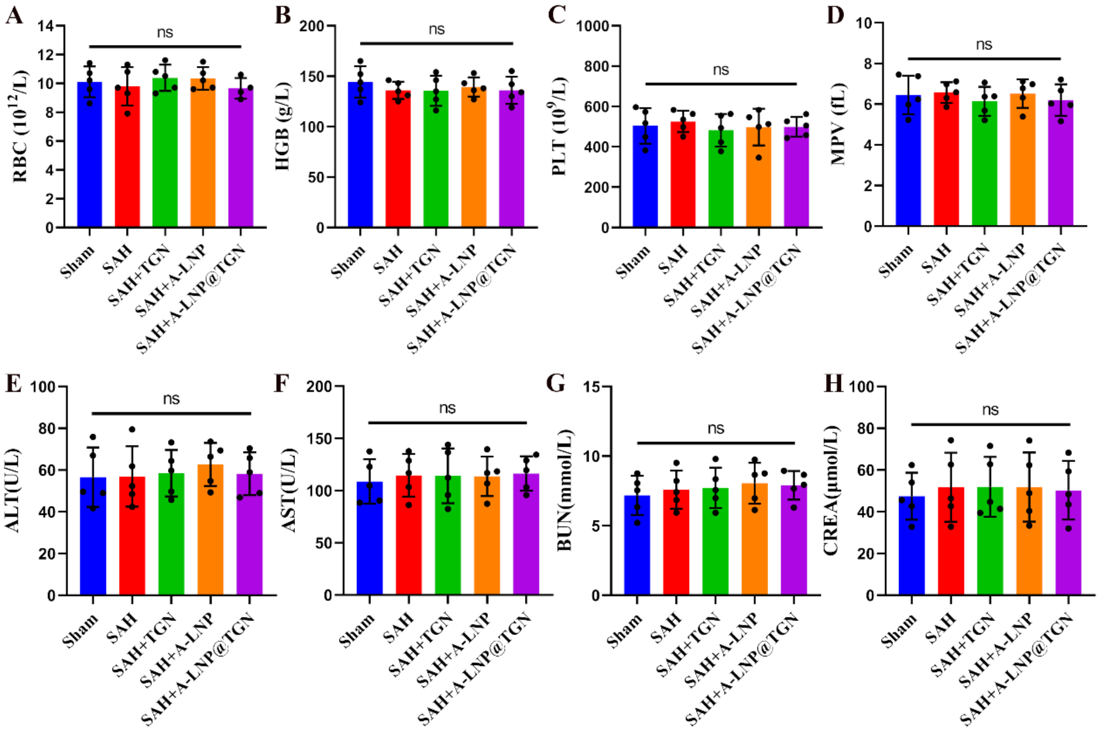

Figure S6. A-D) Blood routine examination of RBC, HGB, PLT, and MPV in mice in each group after different treatments (n = 5). E-H) Serum biochemistry analysis of ALT, AST, BUN, and CREA in mice in each group after different treatments (n = 5). Data are presented as the means ± SD, ns = no significance.

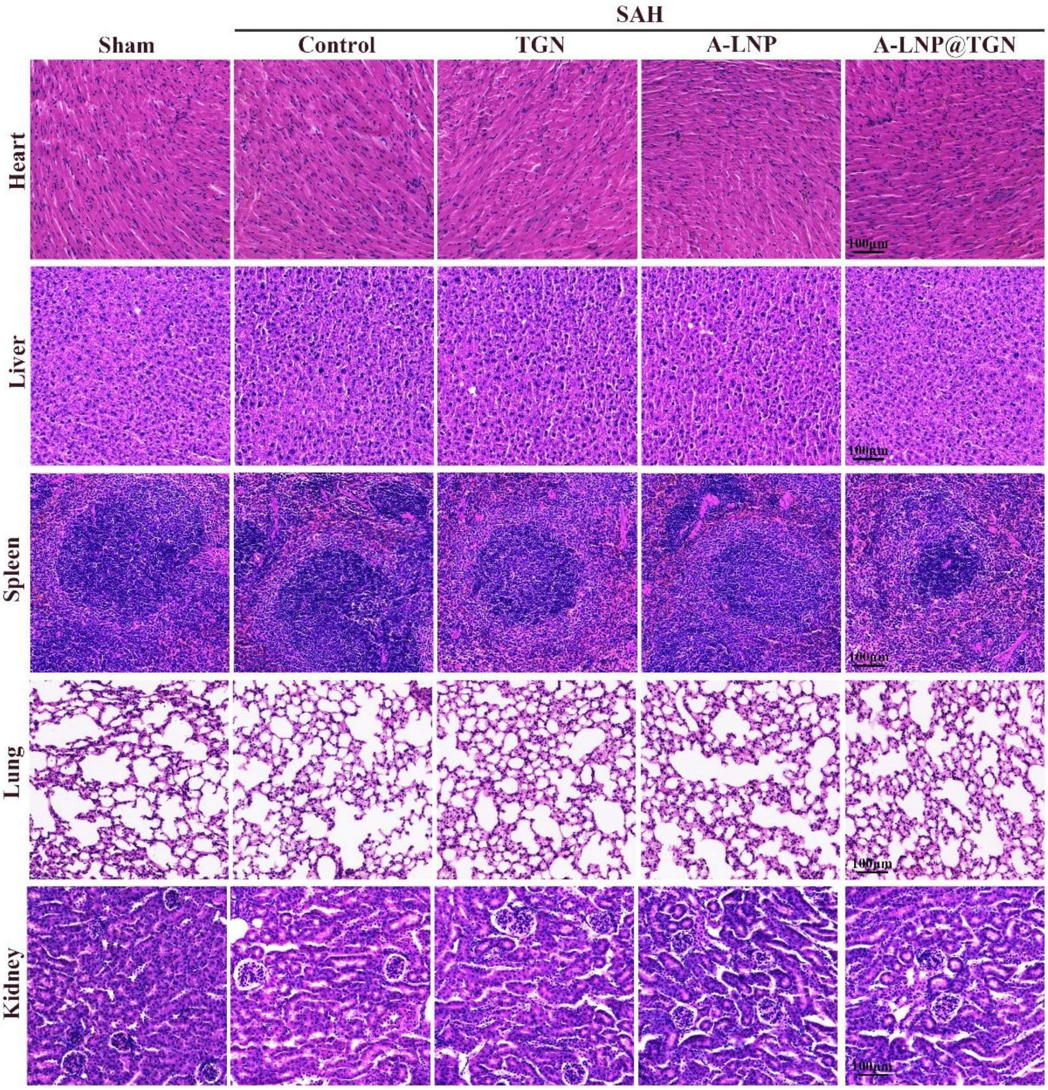

Figure S7. Representative images of hematoxylin-eosin staining of the main organ after SAH-induction with different treatments (n = 5).

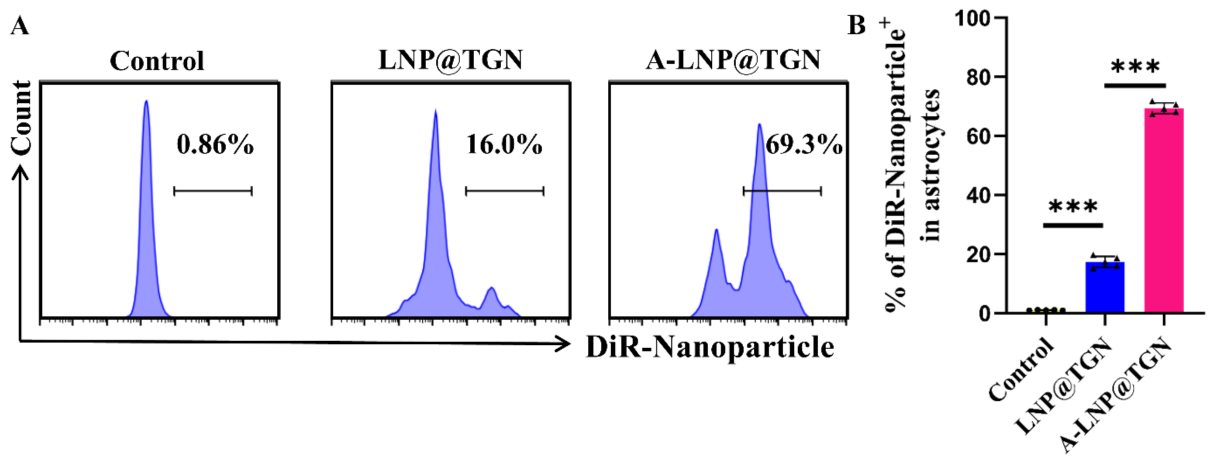

Figure S8. A) Representative images of flow cytometry of the cellular uptake by astrocytes with different treatments. B) Corresponding statistical result (n = 5), Data are presented as the means ± SD, ***P ＜ 0.001.

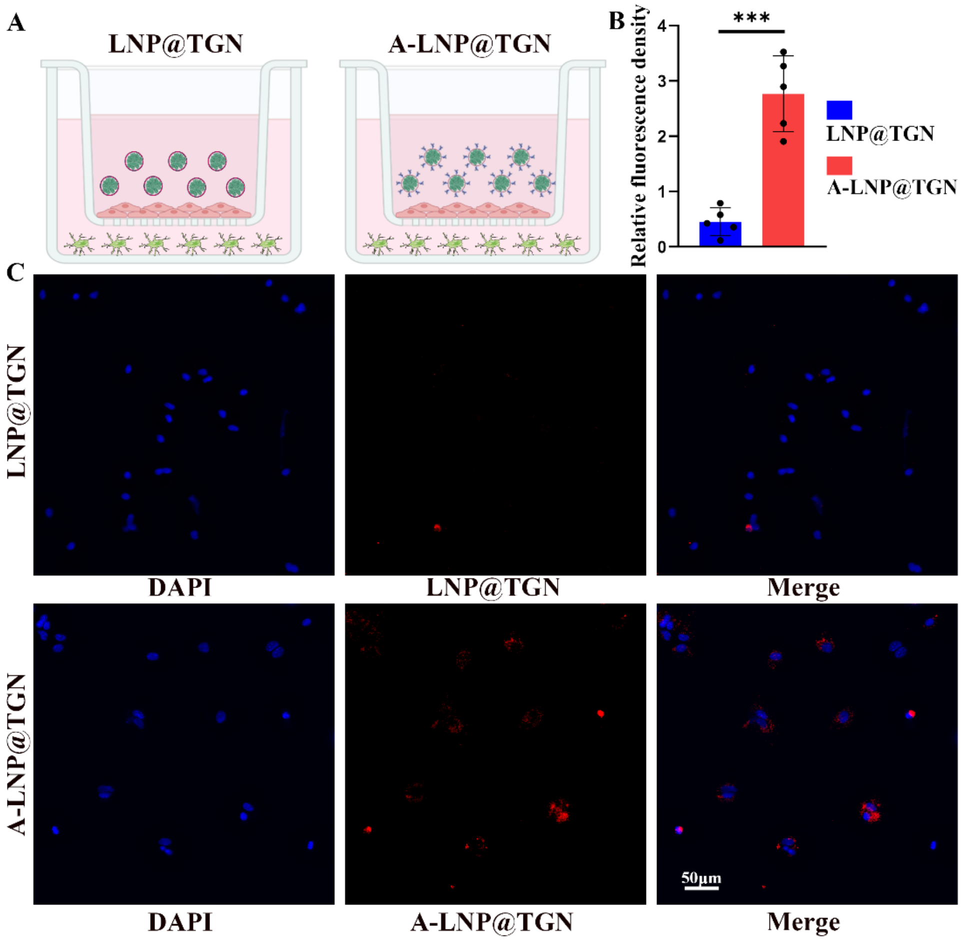

Figure S9. A) Schematic diagram of detecting BBB penetration of A-LNP@TGN. Primary astrocytes were cultured in the lower compartment of the culture plate. The bEnd.3 cells were cultured in Transwell. B) Corresponding statistical result (n = 5). C) Representative immunofluorescence images of the uptake of LNP@TGN and A-LNP@TGN by astrocytes. Data are presented as the means ± SD, ***P < 0.001.

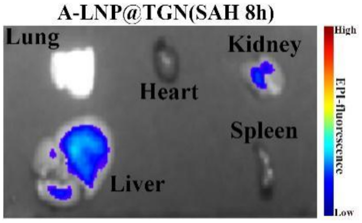

Figure S10. Representative image of in vivo distribution of A-LNP@TGN in main organs at 8 hour after SAH (n = 5).

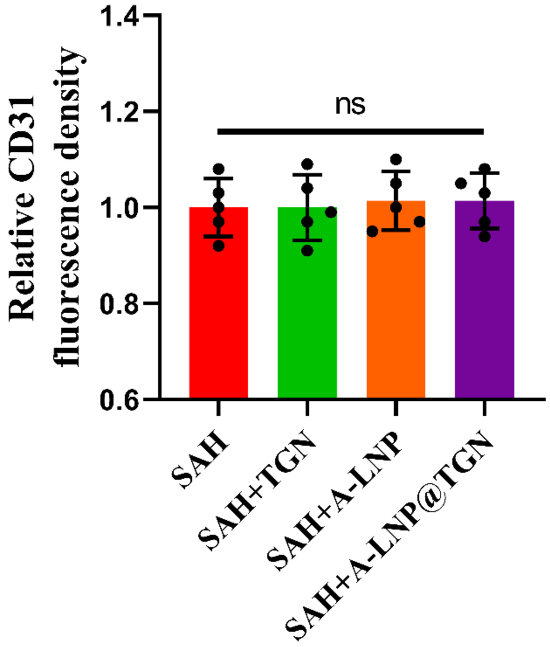

Figure S11. Statistical result of CD31-labeled vascular density in different groups after SAH surgery (n = 5). Data represent the mean ± SD, ns = no significant.

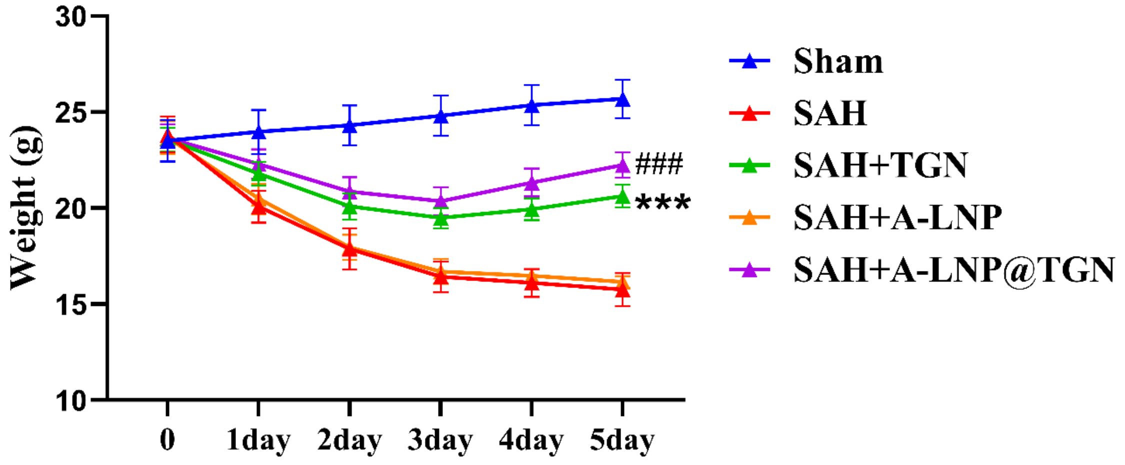

Figure S12. Body weight change in mice with different treatments after SAH surgery (n = 8). Data are presented as the means ± SD. ***P ＜ 0.001 vs SAH, ###P ＜ 0.001 vs SAH + TGN.

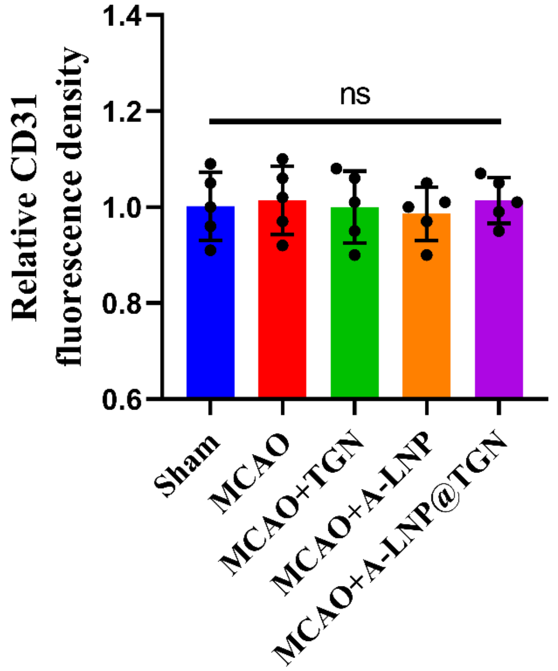

Figure S13. Statistical result of CD31-labeled vascular density in different groups after MCAO surgery (n = 5). Data represent the mean ± SD, ns = no significant.

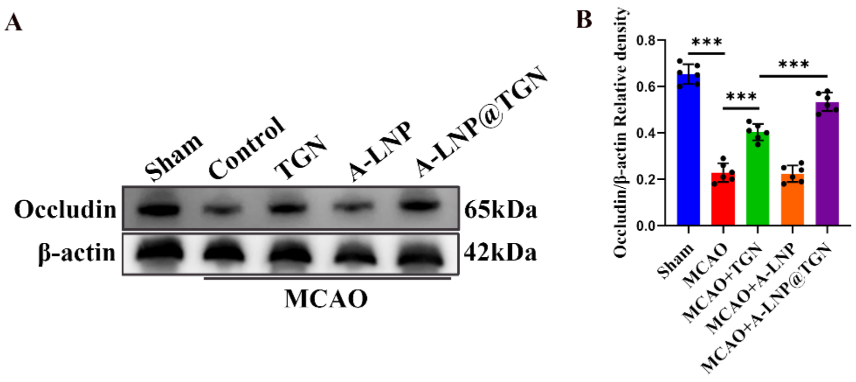

Figure S14. Occludin expression in different groups. A) Representative photographs of western blot. B) Corresponding statistical result (n = 6). Data are presented as the means ± SD, ***P < 0.001.

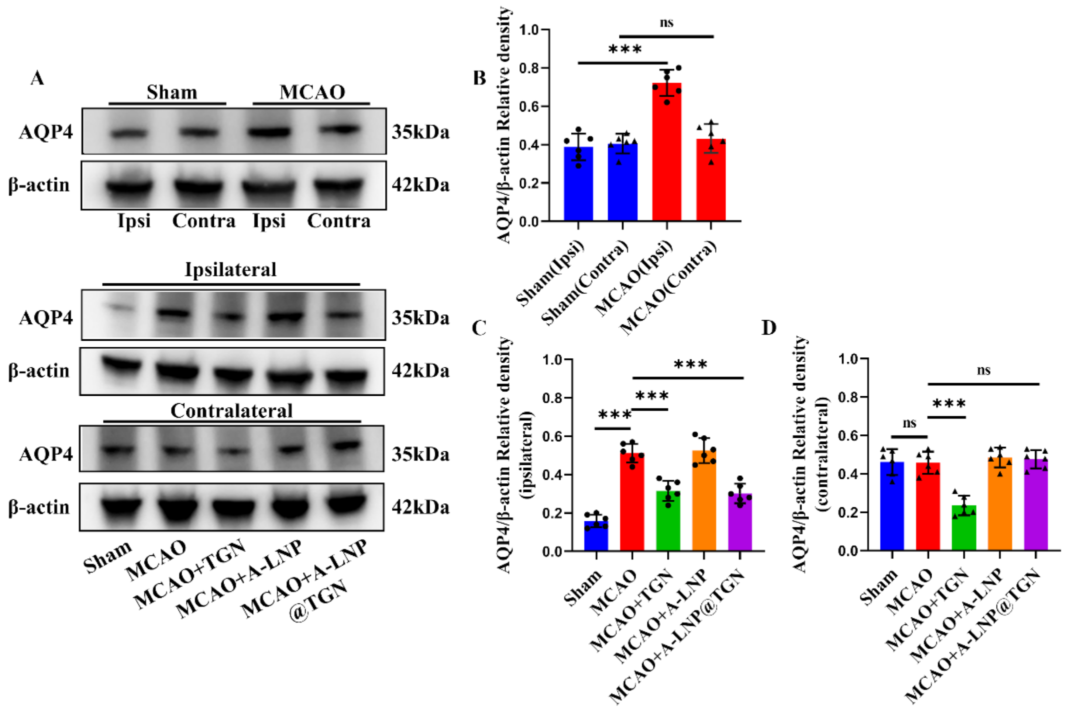

Figure S15. AQP4 expression in different groups. A) Representative photographs of western blot. B-D) Corresponding statistical result (n = 6). Data represent the mean ± SD, ns = no significant, ***p < 0.001.

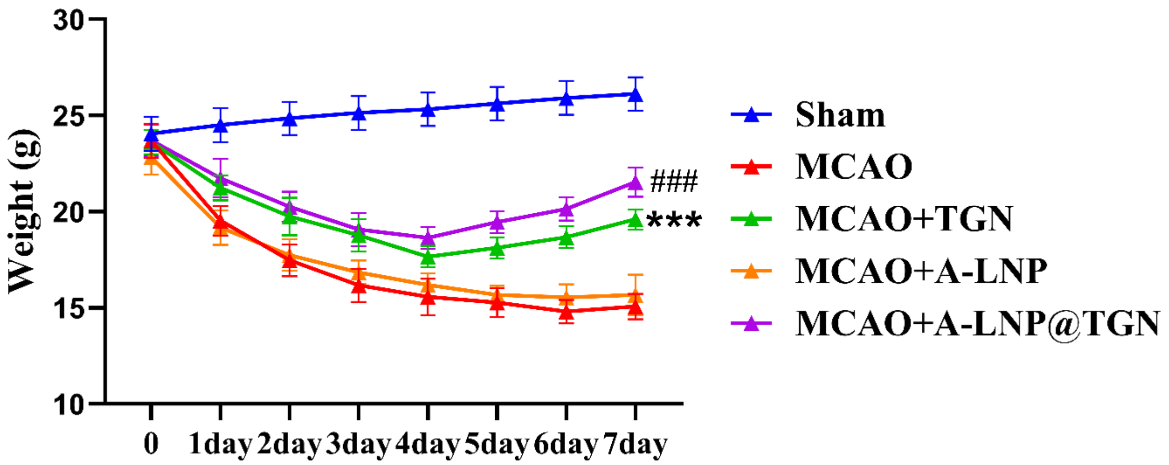

Figure S16. Body weight change in mice with different treatments after tMCAO surgery (n = 8). Data are presented as the means ± SD, ***P < 0.001 vs MCAO, $^{###}$P < 0.001 vs MCAO+TGN.

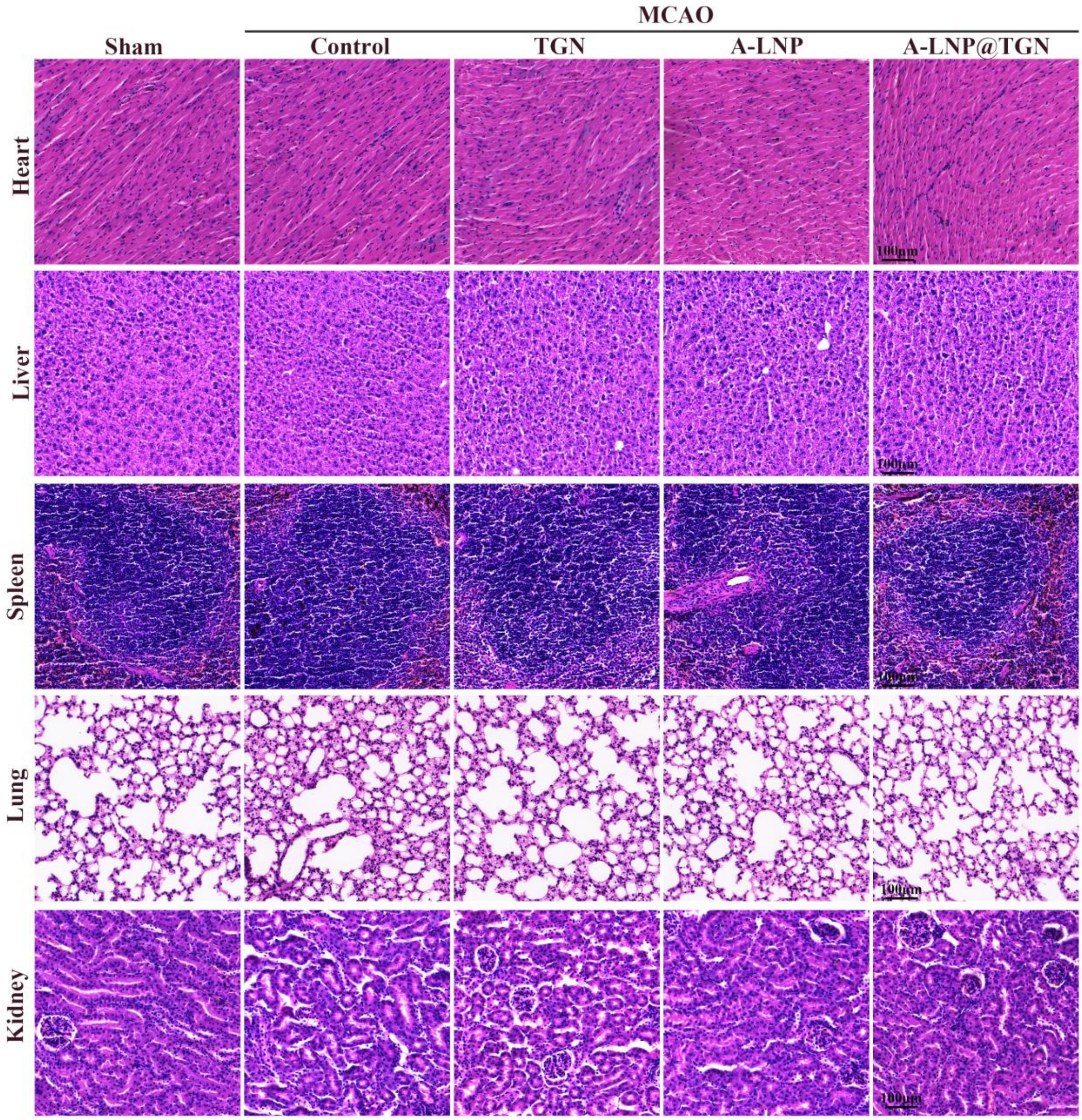

Figure S17. Representative images of hematoxylin-eosin staining of the main organ after MCAO-induction with different treatments (n = 5).

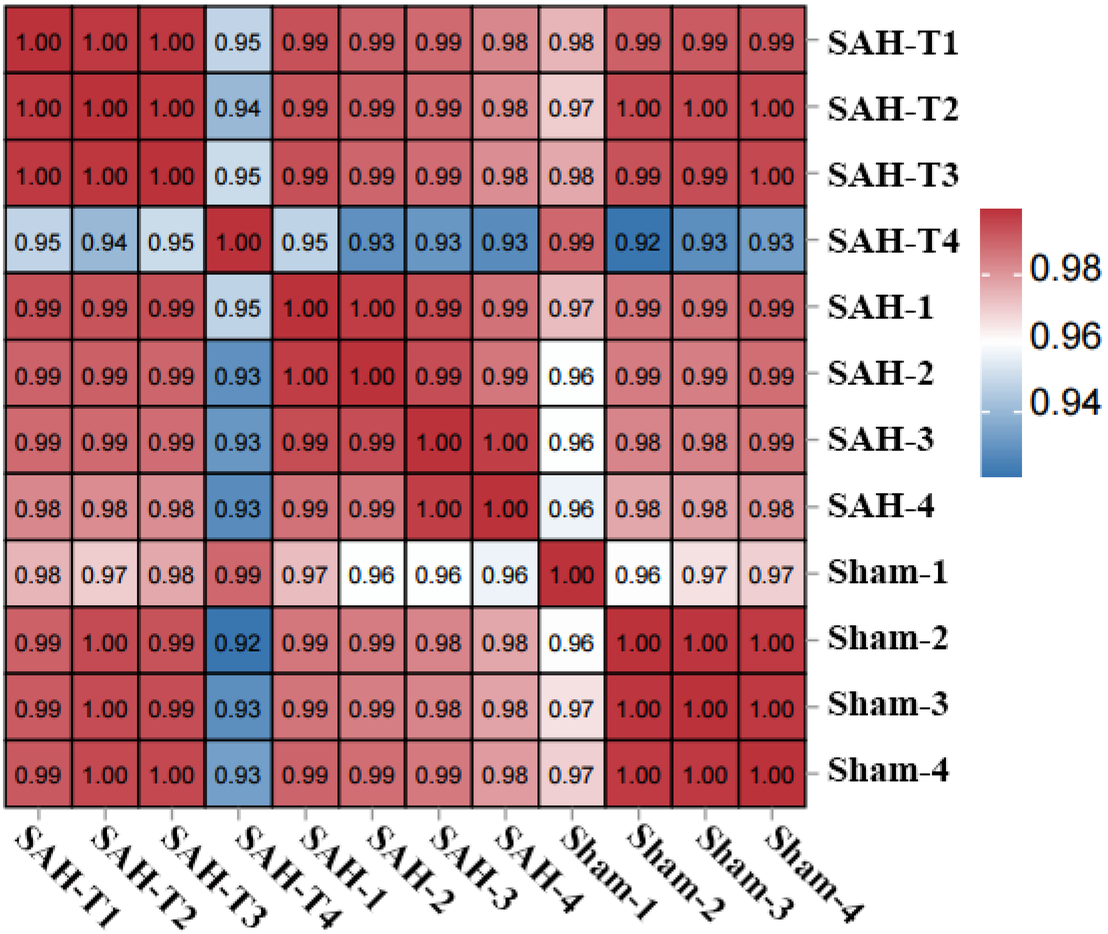

Figure S18. Inter-group sample correlation heatmap of RNA-seq (n = 4). SAH-T = SAH + A-LNP@TGN.

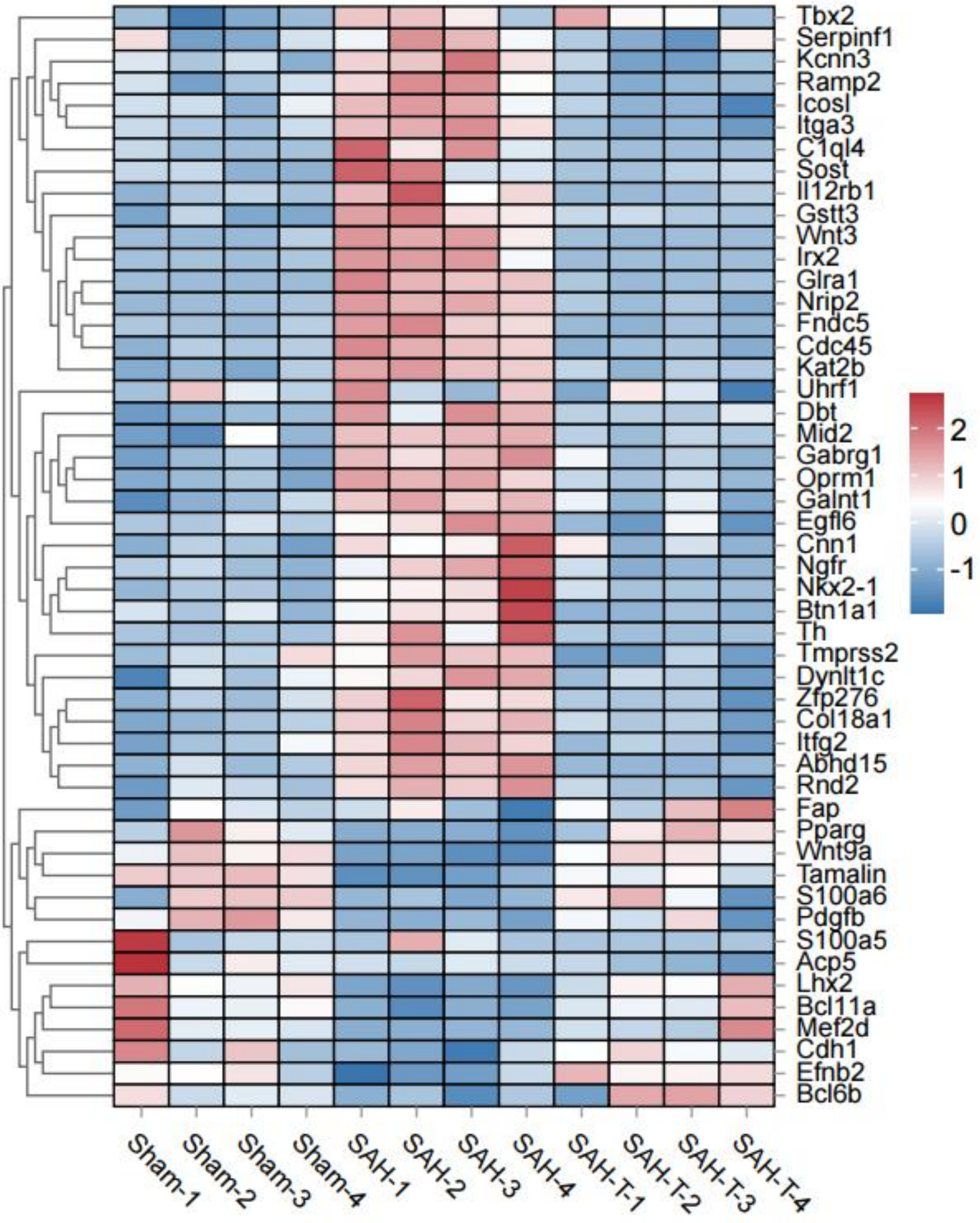

Figure S19. Heatmap of 50 representative DEGs of Sham vs SAH vs SAH with A-LNP@TGN treatment (n = 4).

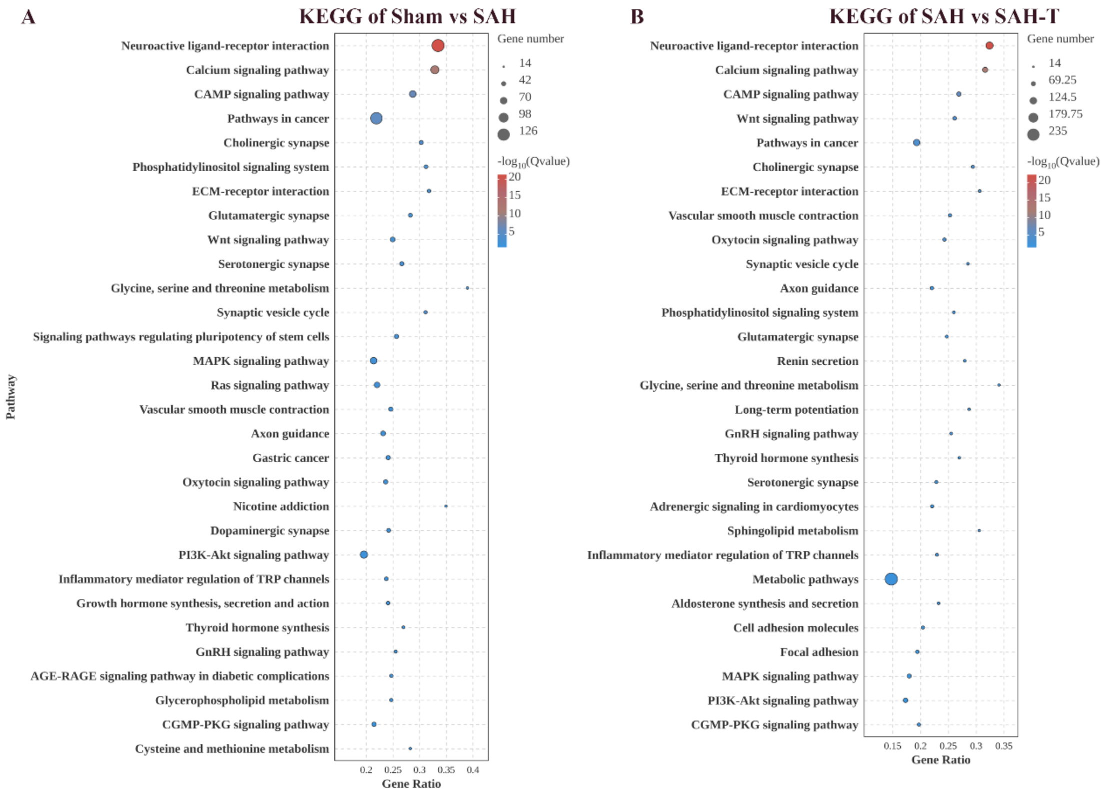

Figure S20. KEGG analysis of DEGs between Sham vs SAH and SAH vs SAH accepting A-LNP@TGN treatment.

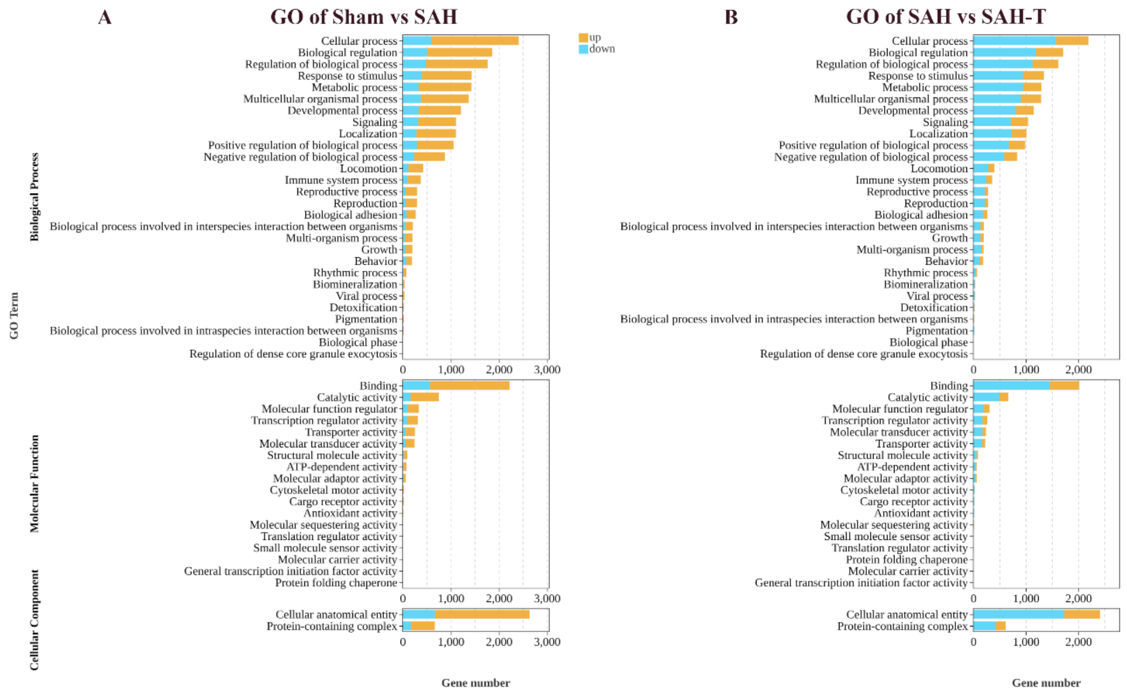

Figure S21. GO analysis of DEGs between Sham vs SAH and SAH vs SAH accepting A-LNP@TGN treatment.

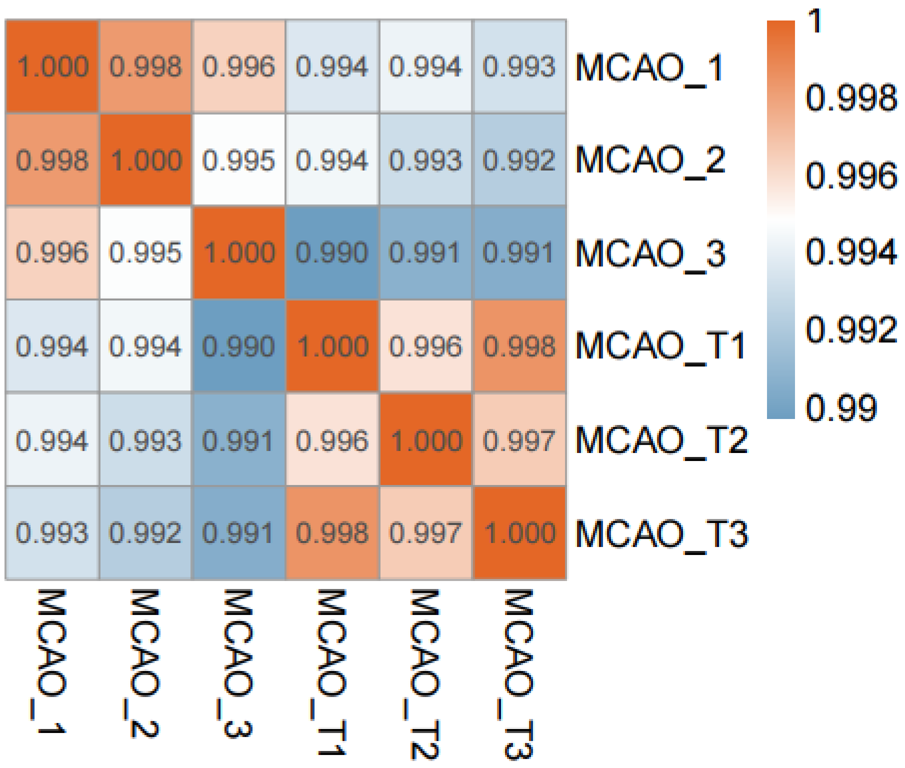

Figure S22. Inter-group sample correlation heatmap of RNA-seq (n = 3). MCAO-T = MCAO + A-LNP@TGN.

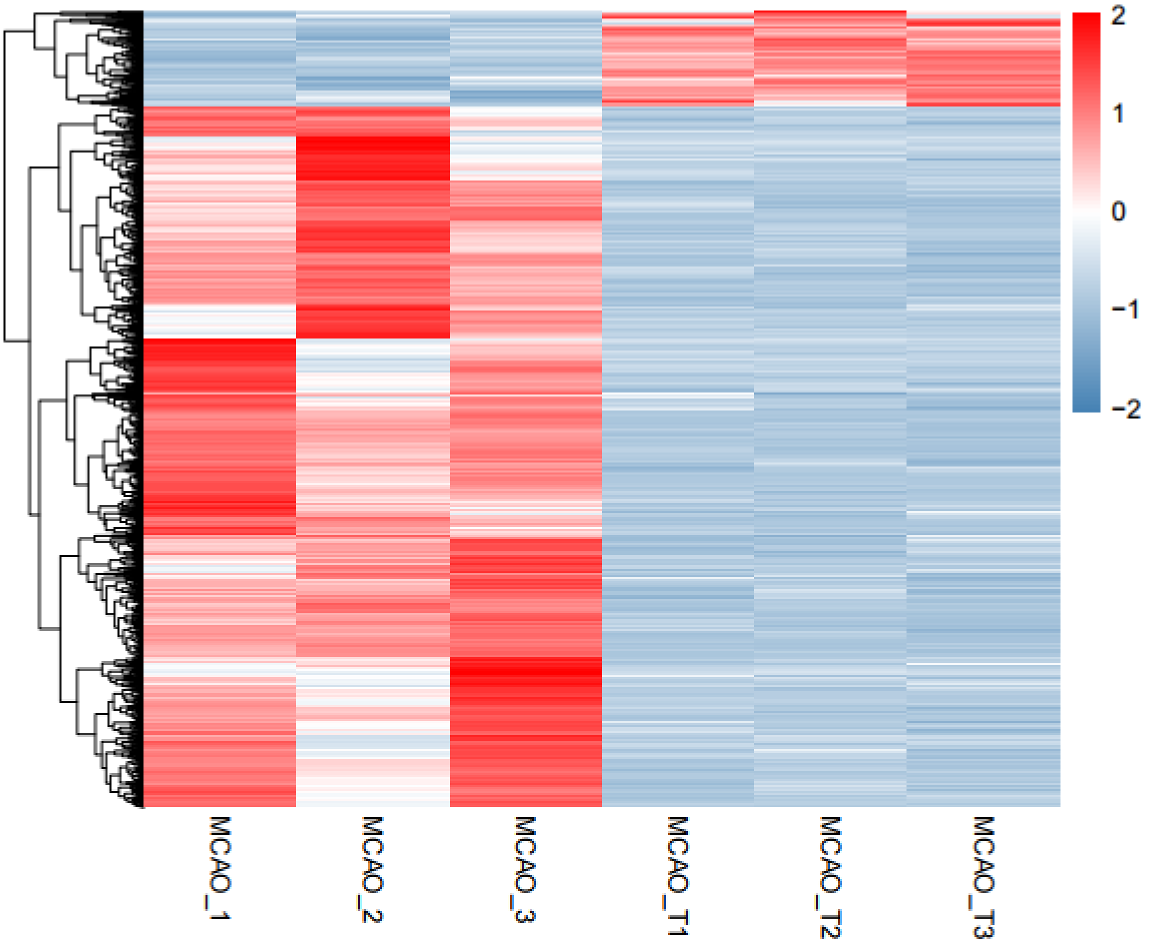

Figure S23. Heatmap of DEGs of MCAO vs MCAO with A-LNP@TGN treatment (n = 3).
